# The antifungal peptide AnAFP from *Aspergillus niger* promotes nutrient mobilization through autophagic recycling during asexual development

**DOI:** 10.3389/fmicb.2024.1490293

**Published:** 2025-01-24

**Authors:** Stephan Starke, Laura Velleman, Birgit Dobbert, Luis Seibert, Jordi Witte, Sascha Jung, Vera Meyer

**Affiliations:** Chair of Applied and Molecular Microbiology, Institute of Biotechnology, Technische Universität Berlin, Berlin, Germany

**Keywords:** *Aspergillus niger*, antifungal protein, nutrient mobilization, asexual development, survival, apoptosis, autophagy, AnAFP

## Abstract

Antifungal peptides are promising drug candidates to fight fungal infections in the clinics and agriculture. However, recent data suggest that antifungal peptides might also play a role within their own producing organism to survive nutrient limiting conditions. We have therefore studied the function of the antifungal AnAFP in *Aspergillus niger* in more detail. To achieve this, we established a Tet-on controlled *anafp* expression system, which allowed us to study a null and an overexpression phenotype in the same isolate. We observed that increased intracellular AnAFP expression reduces growth of *A. niger* and prematurely activates autophagy. Comparative transcriptome analyses of glucose-starving mycelium demonstrated that increased *anafp* expression strongly impacts expression of genes important for cell wall integrity and remodeling, as well as genes with a predicted function in metabolism and transport of carbohydrates, proteins, and lipids. Notably, genes encoding regulators of conidiophore development such as *flbC* and *flbD* became induced upon *anafp* overexpression. Fluorescent analyses of a Tet-on driven AnAFP::eGFP fusion protein congruently unraveled that AnAFP localizes to cell walls and septa of *A. niger*. Moreover, AnAFP::eGFP expression is spatially restricted to selected compartments only and affected cells displayed a sudden reduction in hyphal diameter. From these data we conclude that AnAFP is important to drive vegetative growth and sporulation in *A. niger* during nutrient limitation through autophagic recycling. We predict that AnAFP drives nutrient mobilization through selective cell lysis to ensure the survival of the whole colony during phases of starvation.

## 1 Introduction

The estimated worldwide annual agricultural loss due to pathogenic fungi range from 14 to 18 percent (Savary et al., 2019). Even more threatening is the number of about 1.5 million humans who die each year because of fungal infections (Fisher et al., 2020). To fight the increasing numbers of fungal infections, antimicrobial peptides are discussed as an alternative to traditional drugs and antibiotics, as they have lower risks for resistance development (Cesare et al., 2020). Of specific interests are antifungal peptides from fungal origin which are currently understudied. About 1,400 antifungal peptides are listed in the Antimicrobial Peptide Database (APD3), but only a few dozen are from fungi (Wang et al., 2016; Feurstein et al., 2022; Paege et al., 2016). AFP, the first antifungal peptide from a fungus was isolated in 1965 from the supernatant of the filamentous fungus *Aspergillus giganteus* and served as eponym for other members of the family (Olson and Goerner, 1965; Meyer and Jung, 2018). All peptides of the AFP family are structurally highly conserved and are secreted by filamentous fungi, likely to fight competing fungi in their vicinity. Many of these peptides undergo processing after a signal peptide is cleaved, before maturing to their active form. They are small (about 50 amino acids), net positively charged, and enriched in the amino acids glycine, cysteine and lysine. They contain a specific cysteine spacing pattern that form 3–4 disulfide bridges thought to contribute to their resistance against denaturation or proteolytic degradation. Remarkably, all AFPs are amphipathic which enable them to interact with fungal plasma membranes, although it is thought that the integrity of the plasma membranes of the respective producing fungus remains unaffected (Paege et al., 2019; Hagen et al., 2007; Struyfs et al., 2021). Ultimately, this interaction can either lead to the loss of membrane integrity or the peptides are taken up by the cell to induce regulated cell death. AFP targets can be glucosylceramides, while binding sites for cell wall polymers, i.e., chitin were also reported (Paege et al., 2019; Hagen et al., 2007; Struyfs et al., 2021). However, the specific target(s) for many AFP members remain(s) unknown.

Despite the strong structural similarities among the AFP family members, attacked fungi activate different defense mechanisms to fight their growth inhibitory effect (Meyer and Jung, 2018). For example, the cell wall integrity and the calcium/calcineurin pathway that both lead to the synthesis of cell wall polymers such as glucans and chitin are important to survive attacks by AFP and AnAFP from *A. giganteus* and *A. niger*, respectively, whereas the G protein and cAMP/PKA pathway that leads to membrane polarization, rapid calcium influx, and apoptosis-like cell death is fundamental to survive the attack by PAF from *Penicillium chrysogenum* or NFAP from *Neosartorya fischeri*, respectively.

Notably, AFPs are under tight temporal and spatial expression control in their own producing strains, with a gene expression profile which is similar to that of autophagic genes. Furthermore, expression of AFPs becomes strongly upregulated during adverse conditions including nutrient limitation, osmotic, oxidative, or pH stress but not necessarily in the presence of a competing fungal organism (Meyer and Jung, 2018). The genes are expressed in axenic cultures and conceptually mirror the very tight expression pattern and growth inhibitory activity of cannibal-toxins in endospore forming bacteria (Meyer and Jung, 2018). We thus recently proposed that AFPs could play an endogenous role for their producing organisms comparable to bacterial cannibal toxins, i.e., they sacrifice a subpopulation of its producing mycelium to recycle nutrients under nutrient-limiting conditions (Meyer and Jung, 2018). In agreement, studies on PAF from *P. chrysogenum* and AFPB from *P. digitatum* demonstrated that these AFPs induce cell death of their host cells (Kovács et al., 2014; Bugeda et al., 2020). Also, two meta-transcriptomic analyses in *A. niger* uncovered that the *anafp* co-expression network, which consists of about 600 positively and 400 negatively co-expressed genes, is enriched in gene functions driving processes such as asexual development, polysaccharide catabolism, carbon starvation, nutrient recycling, autophagy, autolysis, cell death, and asexual development (Paege et al., 2016; Schäpe et al., 2023). Proven regulators of *anafp* expression in *A. niger* are so far CreA (carbon catabolite repressor), VelC (activator of secondary metabolism) and StuA (repressor of asexual development) (Paege et al., 2016; Schäpe et al., 2019). Interestingly, regulation of *anafp* expression was predicted to be downstream of *flbA-E* genes (the signaling cascade that activates the asexual developmental transcription factor BrlA) but upstream of the *brlA* gene (Meyer and Jung, 2018).

To better understand the endogenous function of AnAFP in *A. niger*, we harnessed in this study the doxycycline-responsive conditional Tet-on expression system, which is well established for *A. niger* (Meyer et al., 2011; Wanka et al., 2016a; Cairns et al., 2023). We generated a strain in which *anafp* expression is off (no doxycycline addition to the growth medium) or switched on (addition of doxycycline). The impact of AnAFP on the growth of *A. niger* was determined and autophagy quantified in the presence or absence of *anafp* expression. The effect of *anafp* expression was further investigated using RNA sequencing, differential gene expression and gene ontology analyses. Finally, we tracked the intracellular localization of natively expressed AnAFP fused to the green fluorescent protein eGFP using confocal microscopy and live cell imaging.

## 2 Materials and methods

### 2.1 Strains, growth media, cultivation, and storage

All strains used in this study are listed in Table 1. *E. coli* TOP 10 (Invitrogen) was used for cloning. *A. niger* was cultivated in minimal medium (MM) or in complete medium (CM) as described previously (Paege et al., 2016). All *A. niger* strains were stored at −70°C in 0.9% NaCl and 16.7% glycerol. For the generation of a spore solution, CM agar plates were inoculated from the cryo stock and spores were harvested after a 3-day cultivation at 30°C. Spores were suspended in 8 mL 0.9% sodium chloride solution which was filtered through a Miracloth filter. Spore solutions were always made fresh and were stored a maximum of 3 days at 4°C in darkness, prior to inoculation.

**Table 1:** Strains used in this study.

Name	Relevant Genotype (comment)	Genomic locus altered	References
N402 (ATCC64974_4490)	*cspA1-* (wild type)		Bos et al. (1988)
AB4.1	*pyrG-* (N402 derivative)		van Hartingsveldt et al. (1987)
MA170.27	∆*anafp* (AB4.1 derivative)		Wanka et al. (2016a)
FW35.1	*pyrG^+^* (AB4.1 derivative)		Wanka et al. (2016b)
VG8.27	Tet-on*::luciferase, pyr*G*^+^* (AB4.1 derivative)		Meyer et al. (2011)
PK2.9	*Panafp::luciferase, Δanafp, pyr*G*^+^* (AB4.1 derivative)		Paege et al. (2016)
STS4.10	Tet-on*::5’-UTRanafp::anafp::*T*anafp; pyr*G*^+^* (AB4.1 derivative)	*anafp*	This work
STS9.2	*∆*P*anafp*::*anafp; hygR^+^* (AB4.1 derivative)	*anafp*	This work
STS19.3	*hyg*R*^−^; aopyr*G*^+^;* Tet-on*::5’-UTRanafp::anafp::egfp::*T*anafp* (STS9.2 derivative)	*anafp*	This work
JW1.1	Tet-on*::5’-UTRanafp::anafp::*T*anafp; pyr*G*^−^* (STS4.10 derivative)	*anafp*	This work
JW3.3	*pyr*G*^+^;* P*aopyr*G::*egfp* (JW1.1 derivative)	*pyr*G	This work
LV1.1	P*httA::ergA::*T*hxk*; ∆*atg1* (JW3.3 derivative)	*atg1*	This work
LV2.3	P*httA::ergA::*T*hxk*; ∆*atg8* (JW3.3 derivative)	*atg8*	This work
BBA22.6	Tet-on*::anafp::*T*trp*C, *hyg*R^+^ (MA170.24 derivative)	*pyrG*	This work

The *A. niger* strains STS9.2, STS19.3, LV1.1 and LV2.2 were generated via Cas9-mediated DNA integration; 5 μg of purified Cas9 was preassembled with 65 pmol of *in vitro* transcribed sgRNA (Kwon et al., 2019). Cas9 (fused N-terminally to a 6xHis tag and C-terminally to the nuclear localization sequence KKRKV) was expressed in *E. coli* CZ4.1, using the plasmid pET28a/Cas9-Cys (Addgene #53261). The donor DNAs for BBA22.6, STS4.10, STS9.2 and JW3.3 were assembled via Gibson cloning and the DNAs for STS19.3, LV1.1 and LV2.3 were generated via the modular cloning kit from Addgene (Weber et al., 2011). DNA integration in BBA22.6 was performed via single recombination into the *pyrG* locus of strain MA170.27, using hygromycin as a selection marker. For the generation of STS4.10, the Tet-on promotor was fused to the predicted 5’ untranslated region for *anafp* in *A. niger* CBS513.88 (46 bp) (Delmas et al., 2012; Hagiwara et al., 2016). Additionally, the *trpC* terminator used for conventional expression under the Tet-on promotor was replaced by the native *anafp* terminator (1 kb length) as it includes the 3´-UTR of *anafp*. A split marker approach was used for integration and selection was performed using a recyclable *pyrG* marker gene from *A. oryzae* (Arentshorst et al., 2015b). The *anafp* promotor region (1 kb length) was used as 5’ homology arm and the *anafp* terminator was used as the 3’ homolgy arm. In strain STS9.2, the promotor and the open reading frame (ORF) were deleted via replacement with a hygromycin resistance cassette, with 1 kb homology arms binding 1 kb upstream of the *anafp* promotor and to the *anafp* terminator, respectively. For the generation of strain STS19.3, the donor DNA was introduced in STS9.2 via double crossing over, using the same homology arms as used for STS9.2. The AnAFP::eGFP fusion protein is expressed under the Tet-on promotor fused to the 5´ UTR of *anafp*. For the generation of strain JW1.1, STS4.10 was cultivated on 5-FOA while strain JW3.3 was created through a single recombination event into the *pyrG* locus of strain JW1.1 as described earlier (Arentshorst et al., 2015a). For the development of knock-out cassettes used in strains LV1.1 and LV2.1, a terbinafine resistance cassette of P*httA*::*ergA*::T*hxk* was amplified with 60 bp overhang primers homologous to the regions directly upstream of the start codon or downstream of the stop codon of *atg1* or *atg8*. Standard techniques of molecular biology used in this work were described previously (Meyer et al., 2014; Meurant, 2012). All strains were confirmed by Southern blotting as described previously (Sambrook and Russell, 2001). Probes used for detection are listed in Additional File 1.

### 2.2 Cultivation of *Aspergillus niger* on agar plates

For growth quantification of *A. niger* strains on CM agar plates (90 mm), 10^5^ spores in 5 μL physiological salt (0.9% NaCl) solution were point-inoculated in the center of mixed cellulose ester (MCE) membranes [Millipore S-Pak Filter 0.22 μM, 47 mm] which were placed on 20 mL of solidified CM agar. After 16 h of incubation at either 30°C or 37°C, the membranes were photographed and weighed and transferred to a fresh CM agar plate supplemented with 0, 5, or 20 μg/mL doxycycline. After further incubation for 48 h, membranes were weighed again. Biomass and the colony diameter difference was calculated by subtraction of corresponding values between 16 and 64 h of cultivation. Spores were harvested by mixing MCE membranes with 15 mL of physiological salt (0.9% NaCl) solution and a cotton stick was used to bring the spores into solution. The spore concentrations were counted using a Thoma cell counting chamber and the total amount of spores was divided by the colony diameter of the colony.

### 2.3 Biomass quantification of *Aspergillus niger* in shake flask cultures

To measure the optical density of *A. niger* cultures, the Aquila Biolabs system was used, which records backscattered light in shake flask cultures (Bruder et al., 2016). Cell suspension measurements were taken at 60-s intervals using the CGQuant software (version 3.5) allowing for real-time, automated and non-invasive optical density measurements from the flasks in motion. Control flasks with uninoculated cultivation medium served to monitor background scatter. Given the variability of initial optical density values, including those in control flasks, the first 10 readings (10 min) were averaged and set to zero.

### 2.4 Purification of AnAFP from culture supernatants of *Aspergillus niger* STS4.10

*Aspergillus niger* strain STS4.10 was cultivated at 30°C in 250 mL shake flasks containing 50 mL of CM and 20 μg/mL doxycycline inoculated with 5×10^6^ spores/mL. After 24, 48, 72, and 96 h of cultivation, 30 mL of culture broth were centrifuged (4,000 g, 15 min) and filtered (0.2 μm). The supernatants were applied to an FPLC gradient cation-exchange chromatography with a SikiIIPak TOYOPEARL Sulfate – 650F 5 mL, 8×100 mm column from Tosoh Bioscience. The method did run for 78 min at 1 mL/min with buffer A (50 mM NaOAc, pH4) and buffer B (50 mM NaOAc +1.5 M NaCl, pH4). 0–8 min 100% buffer A, 8–70 min 0 to 100% B as a gradient, 72–78 min 100% B.

Elution fractions containing a single Peak at around 65% buffer B were applied to a gradient reversed phase HPLC using a MultoHigh®Bio-200-C18 5 μ column (125×4 mm), (A) milli-Q water and (B) acetonitrile (both solvents acidified with 0.1% TFA). The method run at a flow rate of 1 mL/min, starting with 2% solvent B for 2 min, increasing 2–49% B between 4–16 min and 49–95% B between 16–18 min, hold 95% B till 20 min and decrease 95 to 2% B till 34 min. Concentrations were calculated from the area under the curve using a previously purified and MS verified AnAFP standard (1 mg/mL) as reference. Mass spectrometry analyses was performed at the Institute of Chemistry, Technische Universität Berlin, with an Orbitrap XL from Thermo Fisher Scientific and electron spray ionization. Before ionization, samples were separated via reversed phase liquid chromatography (Grom-Sil 120 ODS-4- HE, 3 µm). Here, eluent 1 (H_2_O + 0.1% HCOOH) and eluent 2 (acetonitrile+0.1% HCOOH) were used at a flow rate of 0.3 mL/min.

### 2.5 Microscopy

For localization of eGFP to vacuoles in *A. niger* strains JW3.3, LV1.1, and LV2.3, respectively, microscopy coverslips were placed in MM supplemented with 0.003% yeast extract (Ohly) inoculated with 5 mL of 10^3^ spores/mL inside a 6 well plate. Biological duplicates were generated for each strain. After 16 h of incubation at 30°C, starvation and *anafp* expression was induced through media exchange (MM lacking glucose and yeast extract but containing 0 or 20 μg/mL doxycycline). The coverslips were further incubated at 30°C and imaged 4 h after media exchange through differential interference contrast and fluorescence microscopy (green channel) on a Leica DM5000 CS system equipped with a DFC365 FX CCD camera. Six images from each biological sample were captured using a HC PL APO CS2 40x objective with a 1.30 aperture. A minimum of 100 vacuoles across all images were counted.

Confocal laser scanning microscopy (CLSM) was performed on *A. niger* strain STS19.3. 10^3^ spores/mL were cultivated within an 8-well μ-slide (ibiTreat) with each well containing 300 μL of MM. After 16 h of incubation at 30°C, starvation and *anafp* expression was induced through media exchange (MM lacking glucose and yeast extract but containing 0 or 20 μg/mL doxycycline). Immediately after inducing carbon starvation by medium change, automated CLSM was performed hourly for a duration of up to 72 h. Imaging was conducted on a LEICA TCS SP8 microscope, utilizing a 20x objective (HC PL APO CS2 IMM, NA: 0.75) paired with a pump that supplied water as immersion fluid for imaging. Microscopy was performed within a temperature-controlled box at 30°C. The excitation laser was set at 488 nm and the detector was set to wavelengths between 495 nm and 545 nm. Z-stacks up to 250 nm were used to create focus stacks of the fluorescence channel using ImageJ (version 1.53 s), while Helicon Focus (version 8.1.0) was used for stacking bright field images.

### 2.6 Nuclear chromatin condensation assay

Investigation of apoptosis-like cell death phenotype was performed as described recently (Semighini and Harris, 2010). In brief, glass coverslips were coated with 0.01% poly-L-lysine solution (Sigma) according to the instruction provided by the manufacturer. Coated coverslips were UV-sterilized for 30 min, sealed and stored at room temperature until use. They were transferred to 6-well plates and 5 mL of MM, inoculated with spores of STS4.10 and FW35.1 to a final concentration of 10^5^ spores/mL. Samples were incubated at 30°C for 15 h. After incubation, the medium was replaced by fresh and preheated MM containing 0, 5 or 20 μg/mL doxycycline. For positive controls, 0, 5, 10, 100, 500, or 850 mM H_2_O_2_ were used instead of doxycycline. The samples were further incubated for 4 h at 30°C. Afterwards, medium was removed, and 5 mL of fixation solution [5% DMSO, 3.7% formaldehyde, 85% PEM buffer (25 mM EGTA, 5 mM MgSO_4_, 50 mM PIPES)] was added to each well, followed by incubation for 15 min at room temperature. After removing the fixation solution, the coverslips were transferred to new wells containing 5 mL PEM buffer and incubated for 5 min. Buffer was discarded and incubation was repeated twice with fresh 5 mL PEM buffer for each time. Next, coverslips were transferred to new 6-well plates with 5 mL of nuclear staining solution (1,000 ng/mL Hoechst 33258 in ultrapure water) and incubated for 5 min. Nuclear staining solution was protected from light throughout the whole experiment. Afterwards, the solution was removed, and 5 mL of PEM buffer was added, followed by an incubation for 3 min. The buffer was discarded, and PEM buffer incubation was repeated three times. Coverslips were carefully dried and transferred, with the hyphae facing down, onto a drop of 10 μL mounting solution (1× PBS buffer, 50% glycerol, 0.1% n-propyl-gallate) on a glass slide. After removing excess fluid, the slides were sealed with transparent nail polish and stored overnight at 4°C. For each sample, 10 images of different regions of a coverslip were taken. Brightfield and fluorescence images [using the CFP channel (460–500 nm) were recorded as z-stacks (10–30 images per stack)] on a Leica DM5000 CS with the CCD microscope camera Leica DFC365 FX, which was used with the 100x, 1.40 aperture HCX PL APO CS objective. For brightfield images, the exposure time was set to 34 ms, for fluorescence images the exposure time was set to 1 s, each setting with a gain of 1.0. Z stacks were processed in image J. All necessary image analysis steps can be found in the ImageJ macro code available in Additional File 6.

### 2.7 Shake flask cultivation of *Aspergillus niger*

For *anafp* mRNA quantification, *A. niger* strains BBA22.6 and STS4.10 were cultivated in 250 mL shake flask cultures filled with 50 mL CM, containing 5×10^6^ spores/mL, in the presence or absence of 20 μg/mL doxycycline, at 30°C and 250 rpm in a shaking incubator (Infors HT, Multitron II). Strains were grown until the mid-exponential growth phase (for 16 h), the biomass was harvested through filtration (Sartorius cotton filter 3 hw, 45 mm, 8–12 μm), shock frozen in liquid nitrogen and stored at −80°C until RNA extraction.

### 2.8 Bioreactor cultivation of *Aspergillus niger*

*Aspergillus niger* strains STS4.10 and VG8.27 were cultivated in a 5 L bioreactor (BioStat A Plus Sartorius) at 30° C as we have described earlier (Nitsche et al., 2012). The strains were cultivated in four liters CM inoculated with 10^6^ spores/mL, in the absence of doxycycline. The initial pH was set to 5.8 and not regulated throughout the cultivation. After 14 h of cultivation, when fungal growth reached the mid-exponential phase, 7 mL of culture broth were harvested in duplicates and filtered through nitrocellulose membranes (Sartorius 0.45 μM, 50 mm). The membranes were placed on glucose free MM agar plates with 20 mL medium containing 0 or 5 μg/mL doxycycline. An additional nitrocellulose membrane was placed on top of the biomass and pressed down tightly with a sterile pestle to avoid an air interface and thus repress sporulation. After 35 h of starvation at 30°C, biomass was scraped from the membranes and shock frozen in liquid nitrogen and stored at −80°C until RNA extraction.

### 2.9 RNA extraction and qPCR

Biomass samples stored at −80°C were thawed on ice and approximately 200–300 mg of wet biomass was mixed with 1 mL of TRIzol solution (Thermo Scientific) and glass beads in a 2 mL screw cap tube. Cell disruption was performed using a VWR 4-Place Mini Bead Mill Homogeniser, applying three 30-s intervals at maximum speed (5 m/s). In-between, cells were put on ice for at least 1 min. Cell debris were centrifuged at 4 °C for 5 min at maximum speed. Total RNA was purified using the Zymo Research Direct-zol RNA Miniprep Kit (R2050) following the manufacturer’s instructions. To eliminate possible genomic DNA, the DNA-free™ DNA Removal Kit from Thermo Fischer (AM1906) was used. cDNA synthesis was carried out with the RevertAid cDNA Synthesis Kit from Thermo Fischer (K1632). Non reverse transcribed RNA was used to control for gDNA contamination in qPCR experiments. For qPCR, 100 ng of cDNA were used as a template with the Blue S’green qPCR master mix (Biozym). RNA quality for qPCR was verified by agarose gel electrophoresis. Actin was amplified using the forward primer: TGTACCCCGGTATCTCCGAC and the reverse primer: CTCGTCGTACTCCTGCTTGG, *anafp* was amplified using ATGCAGCTCACCAGCATTGCCATC (forward) and TCTGGCAATCGACCGTCTTA (reverse).

### 2.10 RNA sequencing and transcriptome analysis

RNA samples were quantified using Qubit 4.0 Fluorometer (Life Technologies, Carlsbad, CA, USA) and RNA integrity was checked with RNA Kit on Agilent 5300 Fragment Analyzer (Agilent Technologies, Palo Alto, CA, USA). RNA sequencing libraries were prepared using the NEBNext Ultra RNA Library Prep Kit for Illumina following the manufacturer’s instructions (NEB, Ipswich, MA, USA). The libraries were sequenced using the NovaSeq instrument with a minimum of 10 million reads per sample. Sequencing quality was verified using MultiQC (Ewels et al., 2016) and untrimmed reads were mapped against the published genome of *A. niger* ATCC64974 (N402) using STAR (Dobin and Gingeras, 2015). For GO term analysis, differentially expressed genes with a log2 fold change >1 and a *p*-value <0.05 (1349 genes, FDR-corrected, Benjamini-Hochberg method (Benjamini and Hochberg, 1995)) were translated into syntenic orthologs of *A. niger* CBS513.88 (1229 genes) using FungiDB (Basenko et al., 2018). For non-syntenic orthologs, BLASTp was used against the genome of *A. niger* CBS513.88 and only the best hit with a score > 60 was considered as an ortholog (101 genes). To classify genes according to their function, existing gene lists were used to find overlaps among the differentially expressed, syntenic, and non-syntenic genes. Raw RNA Seq data has been deposited under: https://www.ncbi.nlm.nih.gov/geo/, accession code: GSE241846. GO term enrichment analysis for differentially expressed genes responding to *afpB* overexpression in *P. digitatum* was done using FungiFun2 in *P. digitatum* (strain PHI26 / CECT 20796) (Priebe et al., 2015).

## 3 Results

### 3.1 Dynamics of *anafp* expression correlates with growth speed of the *Aspergillus niger* colony

**Figure 1:**
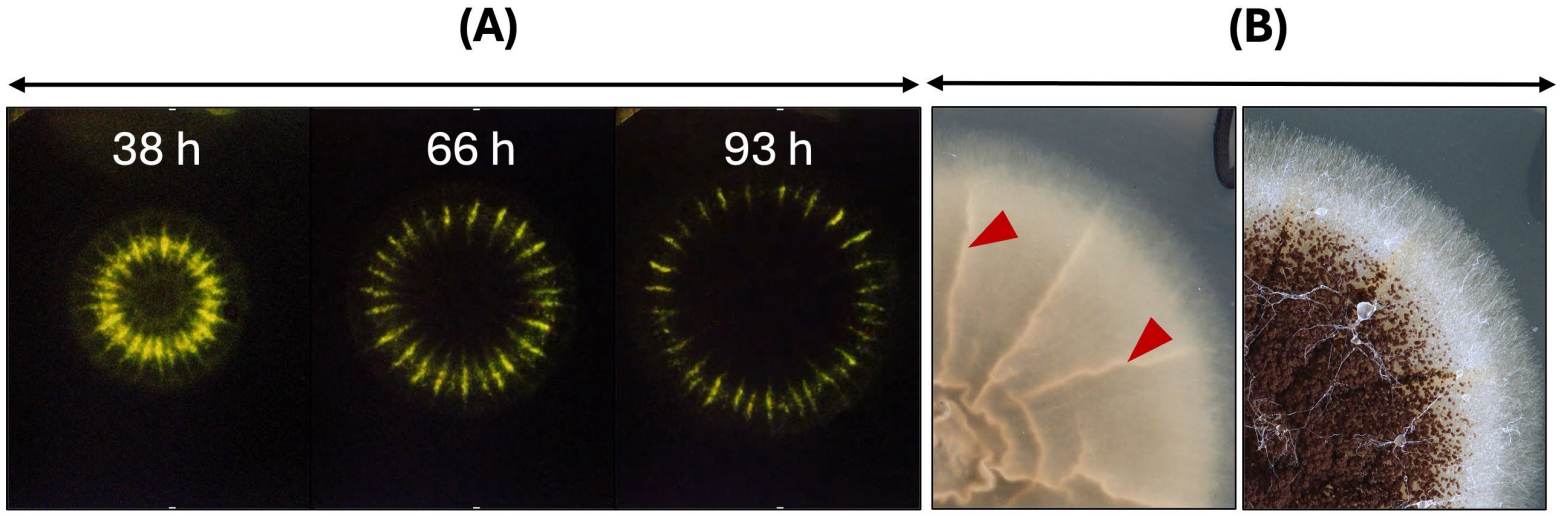
The *anafp* promotor activity within a growing colony of *A. niger* on solid CM agar. **(A)** The promotor activity of *A. niger* PK2.9 (P*anafp*-*luciferase*; Δ*anafp*) is visualized through the luciferase reporter gene, which uses medium-supplemented luciferin as substrate [for cultivation and detection details see Schäpe et al. (2023)]. Three timepoints, derived from a time lapse video of long exposure photographs in darkness, photographed from the bottom (see Supplementary Video S1). The luminescence within the colony of *A. niger* appears as a greenish-yellow concentric ring and is enhanced in wrinkled areas of the colony likely due to more condensed biomass expressing the reporter gene. Note that the luminesce signal fades during prolonged incubation time, supposedly due to limited luciferin stability. **(B)** Colony of PK2.9 photographed from the bottom (left) and top (right). Red arrowheads mark wrinkled colony structures, where luminescence is detected as much brighter signal. An overlay of short exposed colonies under light conditions and luminescent long-exposed images can be seen in Schäpe et al. (2023).

Given that the primary function of AnAFP is to protect the extracellular space against nutrient competitors, one would expect highest *anafp* promoter activity at hyphal tips of foraging hyphae at the colony’s edge. To visualize the dynamics of the *anafp* promoter activity, we thus used our previously described *A. niger* strain PK2.9 in which the *anafp* gene has been deleted and the *anafp* promoter drives expression of the reporter gene luciferase *anafp* instead (Paege et al., 2016). This strain was cultivated on complete medium (CM) agar in the presence of luciferin to resolve the *anafp* promoter activity spatially and temporally through long-exposure photography. Supplementary Video S1 and Figure 1 illustrate that the luminescent signal forms a concentric ring along the outer rim of the colony that continuously moves with the same speed as the growth speed of the colony. Most notably, the concentric *anafp* expression ring has a thickness of only 1–2 mm and is continuously kept a few mm behind the growth front, i.e., exactly when the vegetative mycelium acquires the competence to form conidiophores (Schäpe et al., 2023). This demonstrates that transcriptional activation of the *anafp* promotor does not occur at the apical growth front but becomes induced when asexual development becomes initiated and shortly thereafter repressed.

### 3.2 5’ and 3’ untranslated regions of *anafp* are fundamental for its gene expression

**Figure 2:**
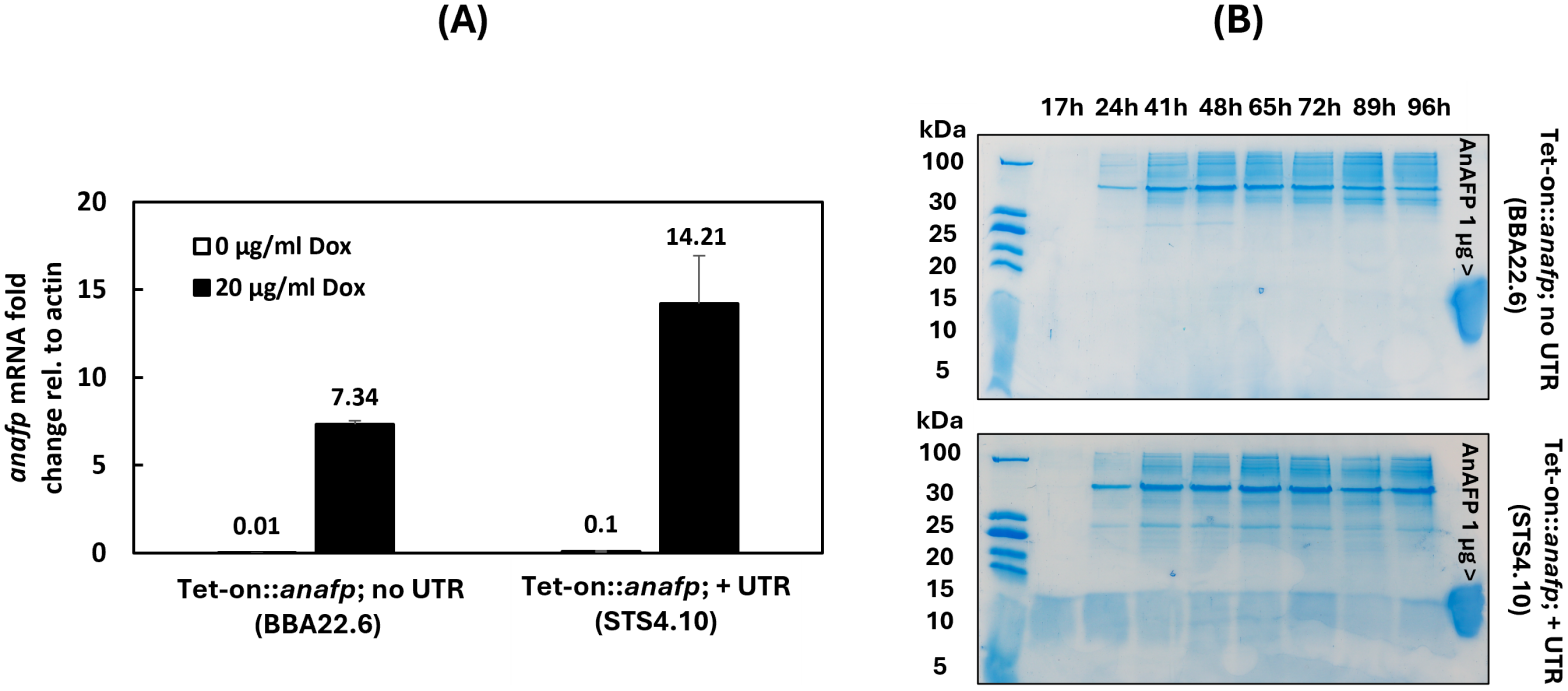
Tet-on driven *anafp* gene expression and the impact of 5′ and 3’ untranslated regions on its translation to AnAFP. **(A)** Quantification of *anafp* mRNA abundance in response to Dox for strain BBA 22.6 (Tet-on::*anafp*; no UTR) and strain STS4.10 (Tet-on::*anafp,* with UTR). **(B)** SDS-PAGE of a 50 mL culture supernatant of strains BBA22.6 (top) and STS4.10 (bottom). The strains were cultivated in CM + 20 μg/mL Dox at 30°C. Culture supernatants were sampled between 17 and 96 h, concentrated 4-times and 20 μL were used for protein gel electrophoresis. 1 μg purified AnAFP was used as positive control. Note that AnAFP protein bands often appear as a smear around 10 kDa.

A conditional Tet-on::*anafp* strain was harnessed to study the effect of *anafp* deletion and overexpression on the growth of *A. niger*. Therefore, the coding sequence of *anafp* was cloned into the Tet-on system (Meyer et al., 2011) and integrated in the previously described strain MA170.27 lacking the *anafp* gene (Wanka et al., 2016a). Integration via homologous recombination at the *pyrG* locus in the resulting strain BBA22.6 was verified by Southern blotting and its capability to transcribe *anafp* mRNA in response to doxycycline (Dox) via qPCR. For cloning details and Southern data see Materials and methods and Supplementary Figure S1. Figure 2A demonstrates that *anafp* mRNA is detectable in large amounts in the presence of 20 μg/mL Dox, while nearly absent when Dox was omitted. However, the AnAFP protein was unexpectedly not detected in the supernatant of shake flask cultures between 17 h and 96 h (Figure 2B). We thus suspected that the presence of the *anafp* 5’ and 3′ untranslated region in the Tet-on system might be of importance for post-transcriptional regulation of the *anafp* gene (Note that usually only the ORF of a gene of interest is cloned in the Tet-on system), where it becomes integrated directly after the minimal promoter of *gpdA* (Pmin) that follows by operator binding site *tetO7* (Meyer et al., 2011). Untranslated regions (UTRs) are positioned in the promotor and terminator regions of eukaryotic genes and often play central roles in the translational regulation of eukaryotic genes (Leppek et al., 2018). We thus determined the UTR sequences of the *anafp* gene from previously published RNA sequencing data (Delmas et al., 2012; Hagiwara et al., 2016; Schäpe et al., 2023) and decided to integrate the Tet-on promoter at the native *anafp* locus, i.e., after the *anafp* promotor region and right before the predicted 5’ UTR sequence (−46 bp upstream of the start codon ATG). The terminator sequence (1 kb, including the 3’ UTR) was left unchanged. For cloning strategy and Southern blot verification, see Supplementary Figure S2. The resulting strain was named STS4.10. Through the integration of the Tet-on::*anafp* expression cassette at the native *anafp* genomic locus, we could detect both Dox-dependent transcription of *anafp* (Figure 2A) and secretion of AnAFP into the culture supernatant of STS4.10, as early as 17 h of cultivation (Figure 2B). The identity and full-length of AnAFP was verified via mass spectrometry (Supplementary Figure S3).

### 3.3 Overexpression of AnAFP reduces growth of *Aspergillus niger*

**Figure 3:**
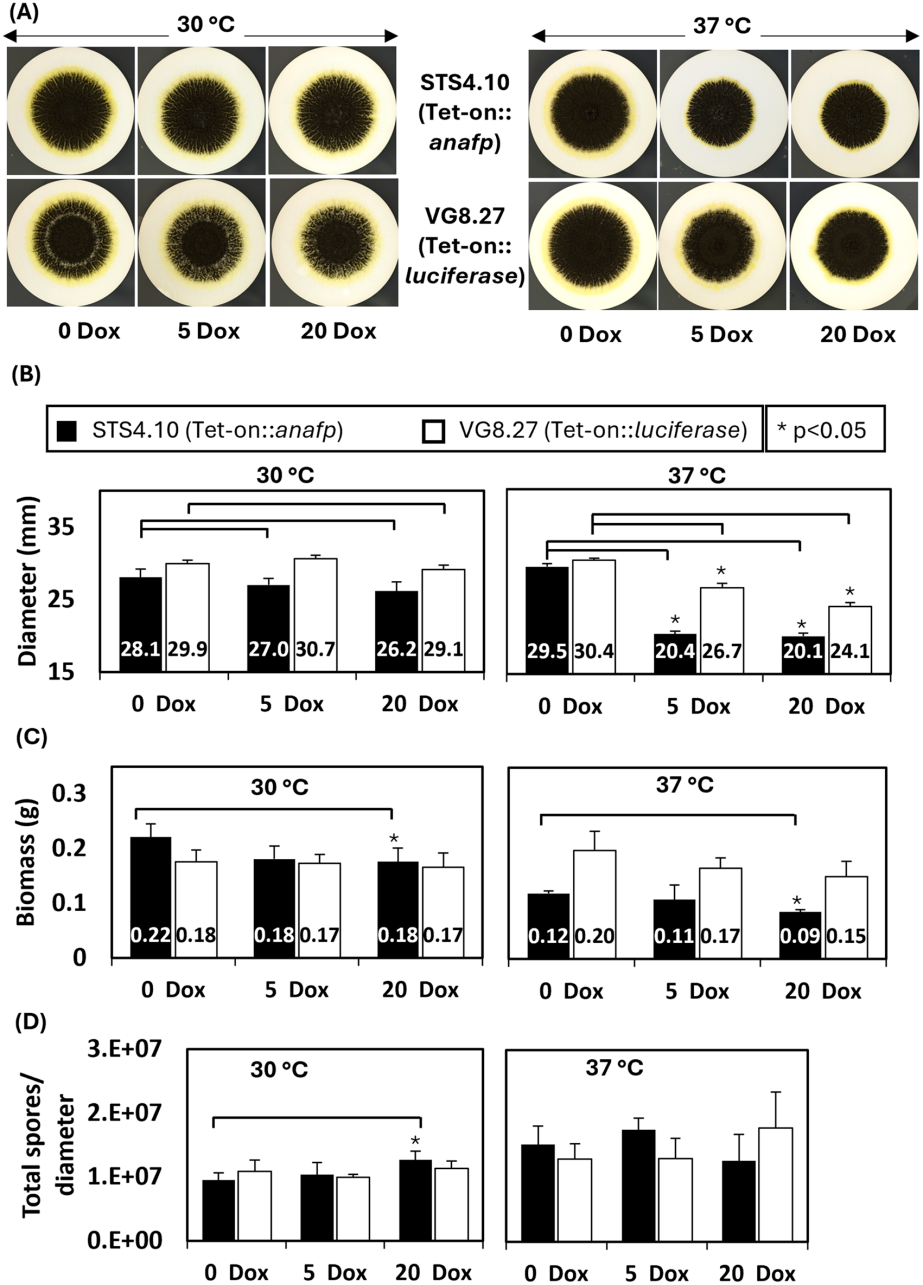
Overexpression of AnAFP reduces growth but not sporulation in *A. niger*. **(A)** Colony of strain STS4.10 (Tet-on::*anafp*) (top) and VG8.27 (Tet-on-*luciferase*) (bottom) on a mixed cellulose ester membrane (0.2 μm) on complete medium agar containing 0, 5 and 20 μg/mL Dox at 30°C or 37°C. After 64 h, colony diameter **(B)**, biomass **(C)** and spore production normalized to the colony diameter **(D)** were determined as described in Materials and methods. Black bars refer to strain STS4.10 and white bars to a control strain VG8.27 which expresses the reporter gene luciferase instead of *anafp* under control of Tet-on (Meyer et al., 2011). Data derive from biological triplicate experiments (*n* = 3), *p*-value derived from paired, two tailed T-test.

**Figure 4:**
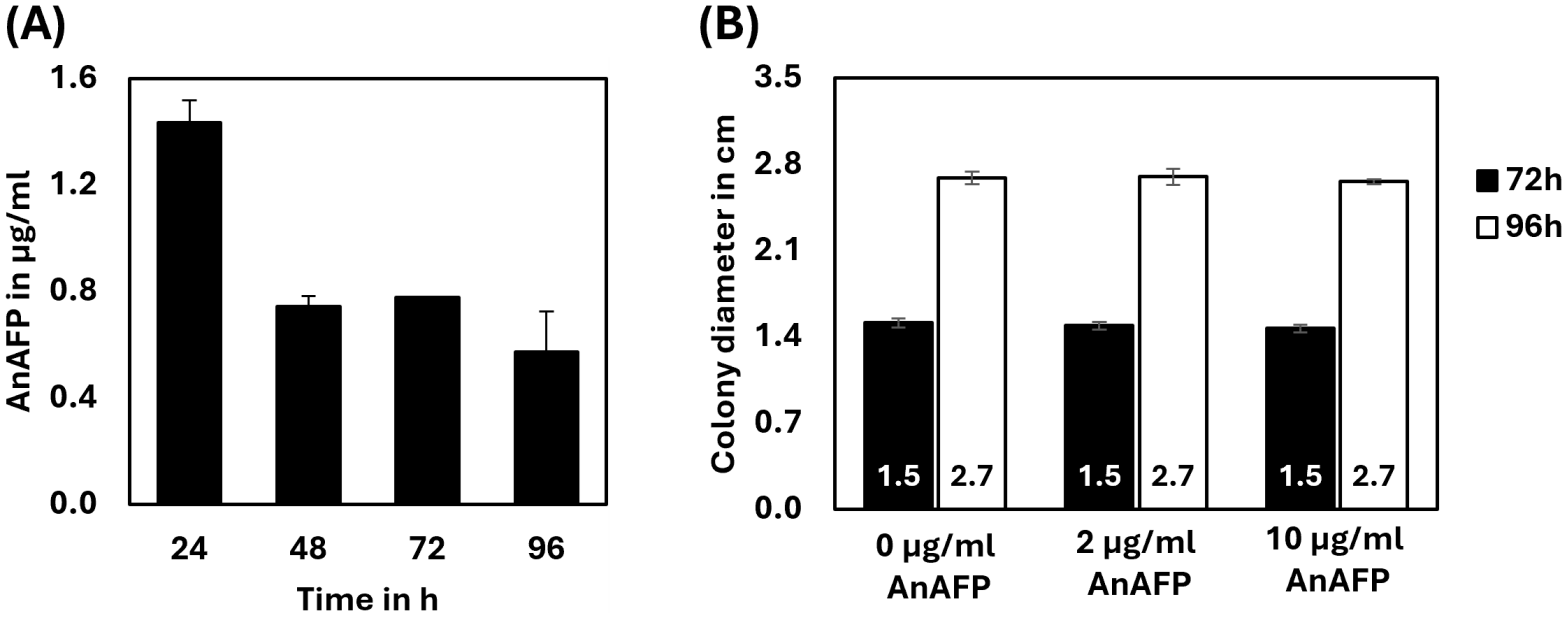
Externally applied AnAFP does not inhibit growth of *A. niger*. **(A)** The amount of AnAFP secreted by STS4.10 in shake flask cultures when cultivated up to 96 h at 30°C (*n* = 2). For cultivation and purification details see Materials and methods. **(B)** Colony diameter of strain STS4.10 after 72 h or 96 h of cultivation in the absence or presence of externally applied AnAFP (0, 2, and 10 μg/mL; *n* = 5).

To study the effects of AnAFP on the growth of *A. niger*, spores of strain STS4.10 (Tet-on::*anafp*) were point-inoculated on complete medium (CM) agar supplemented with 0, 5 or 20 μg/mL doxycycline (Dox) and biomass accumulation was monitored over time. As depicted in Figure 3, colony diameter reduced with increasing Dox concentrations, i.e., increased AnAFP production, an effect that was more pronounced when the cultivation temperature was set to 37°C compared to 30°C. Notably, there was a growth reduction measured in the overexpression control VG8.27 (Tet-on-*luciferase*). However, this reduction in growth was not as pronounced as it was observable in STS4.10 (Tet-on::*anafp*). Additionally, the number of spores produced remained mostly unaffected, except for a slight increase of spores per colony diameter at 20 μg/mL at 30°C. A similar reduction on growth through AnAFP was observed, when strain STS4.10 was cultivated in liquid shake flask cultures (Supplementary Figure S4). With this cultivation approach, we were able to determine the amount of secreted AnAFP by HPLC. A maximum of 1.4 μg/mL AnAFP was detectable after 24 h of cultivation, which declined to about 0.75 μg/mL AnAFP until the end of cultivation (96 h, Figure 4A).

To determine whether the growth-inhibitory effect of AnAFP is due to an extracellular or intracellular mode of action, we added purified AnAFP to the agar medium of STS4.10. Remarkably, even 10 μg/mL externally supplied AnAFP did not reduce colony diameter of the strain (Figure 4B), suggesting that AnAFP produced by strain STS4.10 exerts its growth-inhibitory effect intracellularly.

### 3.4 AnAFP expression is spatially restricted and reduces hyphal diameter of individual cell compartments during carbon starvation

As the data proposed an intracellular activity of AnAFP, we followed its localization by fusing it to the reporter gene *egfp*. Again, we decided to put *anafp::egfp* expression under the control of the Tet-on promoter, to be able to tune its expression from zero to overexpression. As described in Materials and methods and in Supplementary Figure S5, the Tet-on::*anafp::egfp* construct was integrated into the *anafp* locus and the resulting strain STS19.3 was verified through Southern blotting.

**Figure 5:**
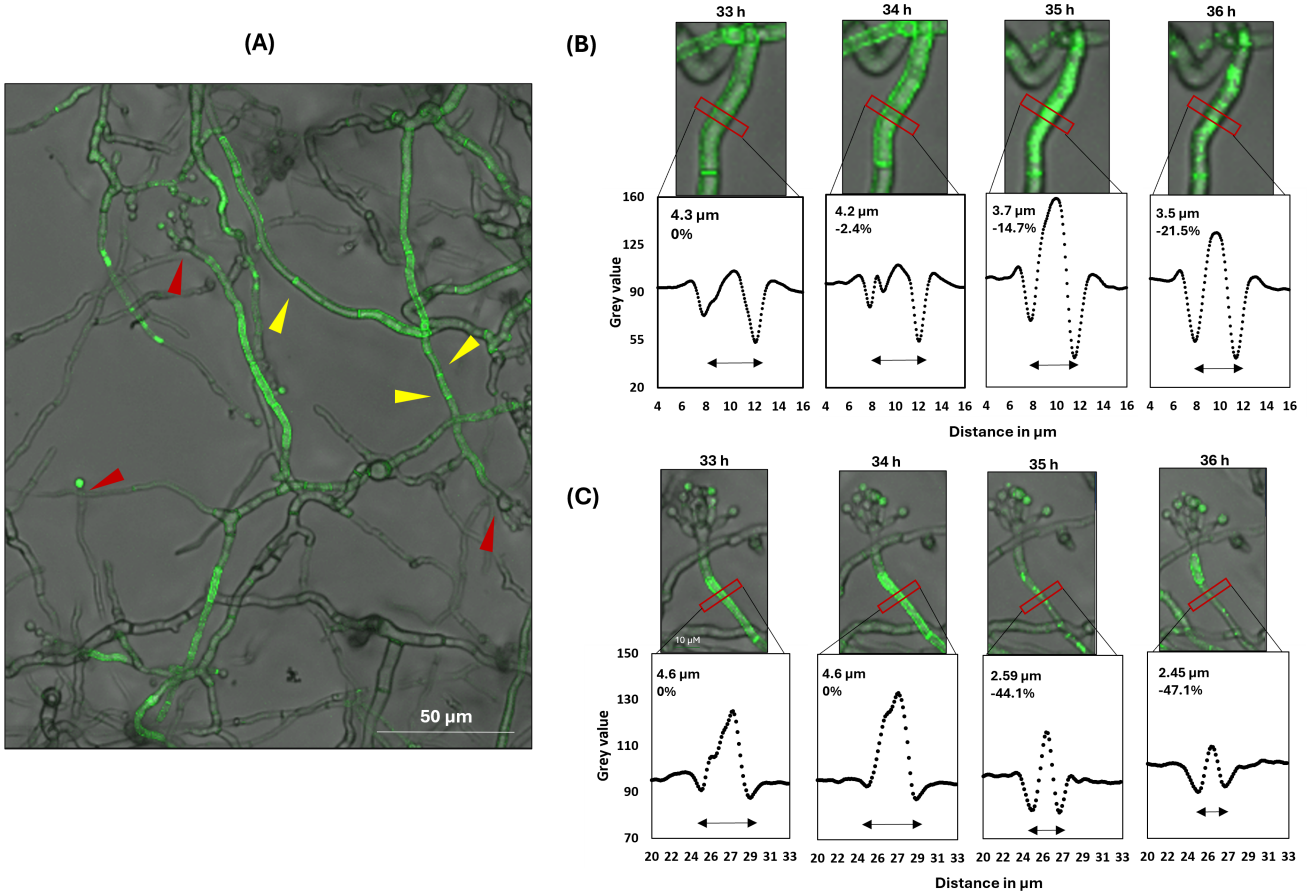
Spatial AnAFP::eGFP localization in strain STS19.3 expressing Tet-on*::anafp::egfp*. Hyphae were cultivated in minimal medium at the bottom of an 8-well microscopy slide. Green fluorescence became detectable only in the presence of 20 μg/mL Dox and after 30 h of carbon starvation. **(A)** The fluorescence signal of AnAFP::eGFP is primarily located in vegetative hyphae and in conidiophores and vegetative hyphae in their vicinity. Conidiophore heads are marked with a red arrowhead, septa with a yellow arrowhead. **(B,C)** show two representative close-ups visualizing the subcellular distribution of the AnAFP::eGFP signal between 33 and 36 h after carbon starvation. The grey values along the horizontal intersection with the hyphae, marked by the red box, is visualized below each micrograph. The hyphal diameter represents the distance between the 2 grey value minima, which estimates the center of the cell wall/ plasma membrane. Corresponding videos are presented in Supplementary Video S2.

Strain STS19.3 was cultivated in 8-well ibiTreat microscopy slides for 16 h in minimal medium (MM) with glucose. After medium exchange to MM lacking glucose and in the absence or presence of 20 μg/mL Dox, growth and fluorescence were monitored hourly via confocal microscopy (see Materials and methods for details). Interestingly, despite being expressed by the Tet-on system in the presence of 20 μg/mL Dox, green fluorescence became only detectable after 30 h of carbon starvation (Figure 5). No fluorescence was measurable in the absence of Dox, in earlier hours after the medium shift or when medium change to MM with glucose was performed (Supplementary Figure S6). Thus AnAFP::eGFP fluorescence only occurred during carbon starvation. Notably, green fluorescence was mainly located at cell periphery and septa of vegetative hyphae, although cytosolic localization was measured as well (Figure 5). Green fluorescence was also detectable in conidiophores and hyphae in their vicinity, whereby neighboring cell compartments differed strongly in their fluorescent signal (Figure 5A). Furthermore, wave-like movement of the fluorescence signal from one cell compartment to the neighboring one was observed (Figure 5C; Supplementary Video S2). Between 33 h and 36 h post-starvation, we noted that cytosolic green fluorescence suddenly agglomerated in individual cell compartments, a phenomenon that coincided with a reduction of their hyphal diameter from about 4.5 μm to about 3.5–2.5 μm (Figures 5B,C). Thereafter, the AnAFP::eGFP signal slowly faded within 1 to 2 h of imaging.

### 3.5 Overexpression of *anafp* leads to a premature onset of autophagy

The sudden reduction of hyphal diameter observed after expression of the *anafp::egfp* fusion protein indicated induced autolysis of selected cell compartments in response to AnAFP. The phenomenon of autolysis of selected cell compartments in response to carbon starvation and the emergence of conidiophores has been previously proposed to be linked to autophagy and asexual reproduction, based on transcriptional data from *A. niger* batch cultures (Nitsche et al., 2012). We thus speculated that induced expression of AnAFP provoked cell death of individual cell compartments under carbon starvation to fuel surviving ones.

In general, cell death in filamentous fungi can be classified into two main categories. The first category is a regulated form of cell death, which can be subdivided into (i) non-lytic (apoptosis, autophagy) (Gonçalves et al., 2017; Hamann et al., 2008; Pollack et al., 2009), (ii) lytic (necroptosis, pyroptosis, ferroptosis, autolysis) (Rico-Ramírez et al., 2022; White et al., 2002; Shen and Naqvi, 2021), and (iii) potentially lytic (HI/VI-mediated cell death, NLR-mediated cell death) (Rico-Ramírez et al., 2022; Uehling et al., 2017). The second category corresponds to a non-regulated form of cell death named necrosis (Gonçalves et al., 2017). Apoptosis and autophagy belonging to the first category are among the most studied cellular processes in fungi, for which a number of biomarkers are known (Sharon et al., 2009). We thus tested the possibility, that AnAFP could potentially provoke apoptosis in *A. niger* and performed a nuclear chromatin condensation assay known as gold standard for apoptosis. However, we could exclude apoptosis as mechanism provoked by AnAFP, as chromatin condensation was not observed upon Dox-induced *anafp* expression, whereas respective control samples did (Supplementary Figure S8).

We therefore tested whether Dox-induced *anafp* expression provoked an autophagic response. This was achieved via genetic modification of strain STS4.10 (Tet-on::*anafp*) that expressed eGFP constitutively in the cytoplasm. We generated this reporter strain as we could show earlier that cytoplasmatic eGFP fluorescence relocates to the vacuole during autophagy (Nitsche et al., 2013). Hence, we assumed that the proportion of fluorescently and non-fluorescently labeled vacuoles of *A. niger* could serve as quantitative proxy for autophagy activity. Cloning and integration of the respective P*aopyrG::egfp*::T*trp*C construct into the STS4.10 background (Tet-on::*anafp*) resulted in strain JW3.3 (for details, see Supplementary Figure S9).

**Figure 6:**
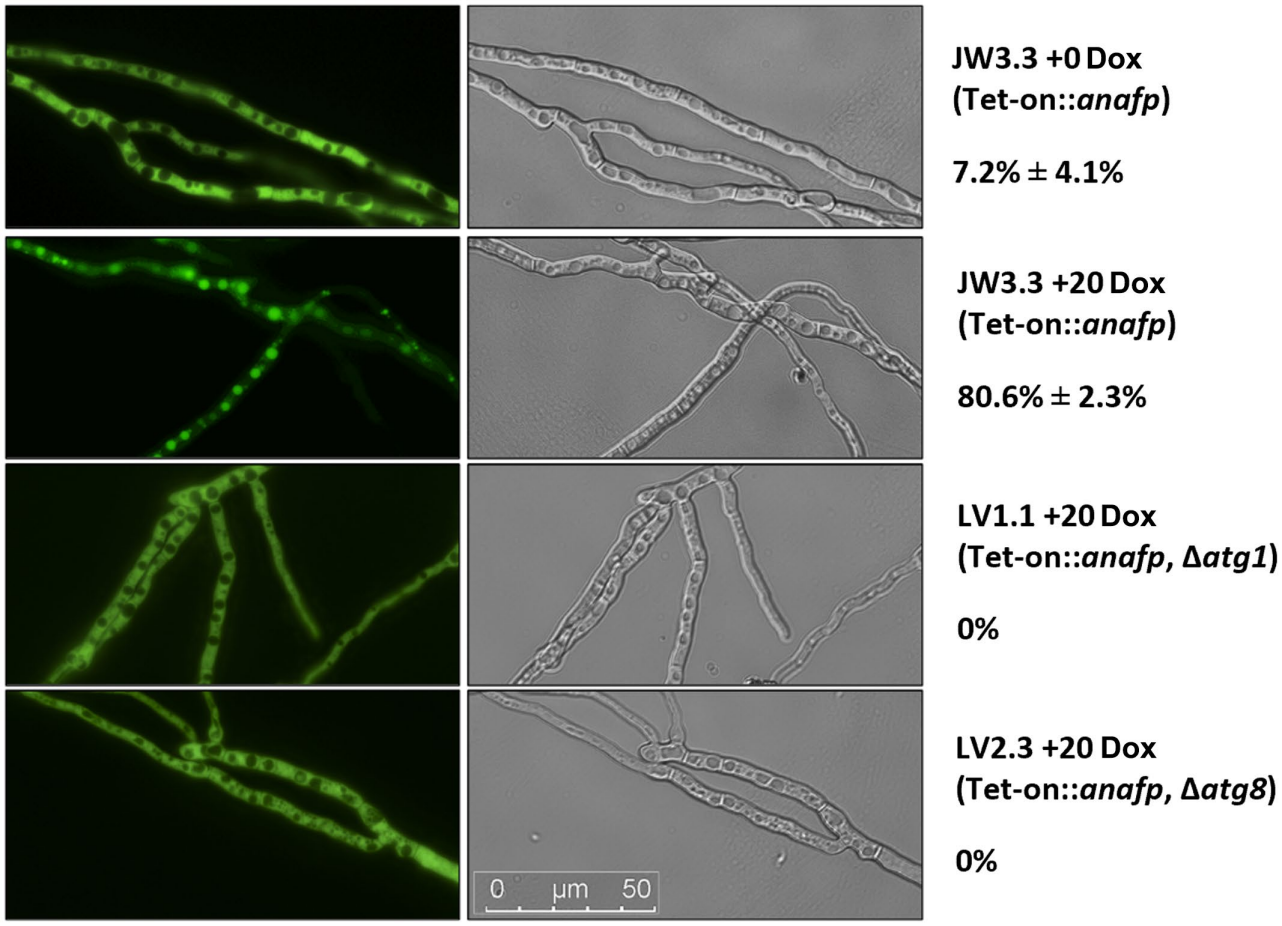
Quantification of autophagy through eGFP localization. Hyphae of strains JW3.3, LV1.1 and LV2.3 were grown on microscopy coverslips and subjected to carbon starvation as described in Materials and methods. Relevant genotype is given below the strain name. For at least 100 vacuoles, fluorescence was measured in the absence (Dox = 0) or presence (Dox = 20 μg/mL) of *anafp* expression. One representative picture is shown of the fluorescence channel (left) and the differential interference contrast channel (right). The percentage of filled vacuoles per total vacuoles counted is plotted next to the respective micrographs. Data refer to biological duplicate experiments.

To determine the initiation and duration of autophagy, spores of strain JW3.3 were placed on microscopy coverslips and cultivated in MM including glucose at 30°C for 16 h, followed by a medium change to MM lacking glucose to induce carbon starvation. Initiation of autophagy in Dox-uninduced hyphae started after 6 h post starvation and already 100% of vacuoles became fluorescently labeled after 10 h post starvation (data not shown). This demonstrated that the autophagic response takes about 4 h in *A. niger,* under the conditions tested. Any impact of *anafp* gene expression on autophagy was thus evaluated after 4 h post starvation. As summarized in Figure 6, only 7.2% ± 4.1% of the vacuoles (*n* = 100) showed fluorescence in the absence of Dox, i.e., when the *anafp* gene was not expressed. In contrast, Dox-induced expression of *anafp* increased the amount of fluorescently labeled vacuoles to 80.6% ± 2.3%, suggesting that AnAFP can provoke autophagy. To verify this assumption, we generated two control strains deficient in: Atg1, a serine/threonine kinase and Atg8, an autophagosome-bound, ubiquitin-like protein. Both genes were shown to be essential for autophagy in *A. niger* (Nitsche et al., 2013). Control strain LV1.1 was a JW3.3 derivative, in which the gene *atg1* was deleted, control strain LV2.3 a JW3.3 derivative, in which the gene *atg8* was deleted. For cloning details and Southern proofs of successful deletion, see Materials and methods and Supplementary Figure S10. As in both strains LV1.1 and LV2.3 Dox-induced expression of the *anafp* gene did not provoke any translocation of the cytoplasmatic eGFP into the vacuoles in contrast to the autophagy-competent progenitor strain JW3.3 (Figure 6), we concluded that the intracellular mode of action of AnAFP is dependent on autophagy (at least of Atg1 and Atg8) and that its overexpression can even induce the onset of autophagy prematurely.

### 3.6 Overexpression of *anafp* provokes nutrient mobilization

Since doxycycline induced expression of AnAFP lead to a reduction in growth and a premature autophagic response, the next step aimed to reveal the underlying transcriptomic network which is promoting these effects. Thus, we determined the genome-wide transcriptional response in strain STS4.10 (Tet-on::*anafp*) during carbon starvation and in the absence and presence of *anafp*. For this experiment, we cultivated this strain in submerged batch bioreactor cultures until the mid-exponential growth phase, harvested the biomass and transferred it to minimal medium agar plates lacking glucose but supplemented with either 0 or 5 μg/mL Dox. Following 35 h of carbon starvation, biomass was isolated from the agar plates, total RNA extracted and subjected to Illumina sequencing as described in Materials and methods. Strain VG8.27 expressing luciferase under control of the Tet-on promoter (Tet-on::*luciferase*) (Meyer et al., 2011) served as a control strain for this experiment and was thus cultivated with the same procedure. With this control we wished to ensure that any observed differential gene expression can be directly linked to *anafp* overexpression and not to an indirect cellular effect due to Dox addition and/or general cellular stress provoked by Tet-on driven protein expression. Differential gene expression analysis (see Materials and methods) of Tet-on driven luciferase expression in control strain VG8.27 (5 μg/mL Dox compared to 0 μg/mL Dox) indeed yielded only 7 genes, demonstrating that Dox addition and/or protein overexpression did not provoke any global transcriptional changes in *A. niger*. However, differential gene expression analysis of Tet-on driven *anafp* overexpression (5 μg/mL Dox compared to 0 μg/mL Dox) yielded a total of 1,349 genes (log 2-fold >1, false discovery rate (FDR) corrected *p*-value <0.05), whereby 599 were upregulated and 750 downregulated (Additional File 2). Notably, *anafp* is one of the most significantly, differentially expressed genes with a log 2-fold change of 8.1 and an adjusted *p*-value of 0.00011 (Additional File 2). As reads were mapped to the *A. niger* N402 genome which is insufficiently annotated, the differentially expressed genes were translated to syntenic orthologs of *A. niger* CBS513.88 which yielded 1,229 unique genes with a log2 fold change >1, which were thus used for GO term enrichment analysis. This analysis uncovered that *anafp* overexpression resulted in a severe up- and downregulation of genes, responsible for the metabolism, hydrolysis and transmembrane transport of carbohydrates, lipids, and proteins (GO:0005975; GO:0006520; GO:0006629; GO:0055085, Table 2; Additional File 3). Notably, 151 of 898 predicted *A. niger* transporters (16.9%) were differentially expressed. Similarly, between 14 and 19 percent of predicted hydrolases (carbohydrates, lipids, proteins) do respond to the presence of *anafp*, a selection of which has been highlighted in Table 3. The enrichment of the GO terms extracellular region (GO:0005576) and plasma membrane (GO:0005886) further underlined the involvement of proteins and enzymes for transport and metabolism upon *anafp* overexpression.

**Table 2:** Gene ontology enrichment of genes differentially expressed in response to *anafp* overexpression in *A. niger* log2 fold change >1, false discovery rate (FDR) corrected *p*-value <0.05; after Benjamini and Hochberg, 1995].

ID	Name	log2FC	*p*-value (FDR)
Biological process			
GO:0055085	Transmembrane transport	1.79	1.70E-09
GO:0005975	Carbohydrate metabolic process	1.84	2.00E-05
GO:0006520	Cellular amino acid metabolic process	1.83	1.60E-03
GO:0006629	Lipid metabolic process	1.54	6.00E-03
GO:0071941	Nitrogen cycle metabolic process	4.71	2.70E-02
GO:0098754	Detoxification	3.44	2.70E-02
GO:0006091	Generation of precursor metabolites and energy	1.83	4.80E-02
Molecular function			
GO:0016491	Oxidoreductase activity	1.93	4.10E-22
GO:0043167	Ion binding	1.45	1.30E-11
GO:0022857	Transmembrane transporter activity	1.8	2.90E-10
GO:0016829	Lyase activity	2.13	3.30E-06
GO:0016810	Hydrolase activity acting on carbon-nitrogen but not peptide bonds	2.41	9.60E-05
GO:0016798	Hydrolase activity acting on glycosyl bonds	1.94	1.90E-04
GO:0008233	Peptidase activity	1.7	9.00E-03
GO:0016757	Glycosyltransferase activity	1.74	3.20E-02
Cellular component			
GO:0005576	Extracellular region	2.53	8.20E-13
GO:0005615	Extracellular space	6.29	6.90E-03
GO:0005886	Plasma membrane	1.51	6.90E-03

**Table 3:** Selected genes responding to *anafp* overexpression and categorized by function.

**Name**	**Differentially expressed/Total number of genes in category**	**Percent**	**Up**	**Down**
***Carbohydrates***	32/170	18.8	13	19
***Proteases***	36/227	15.9	23	13
***Lipases***	9/65	13.8	8	1
***Cell wall remodelling***	14/119	11.8	1	13
***Transporters***	151/891	16.9	76	75
***Transcription factors***	91/691	13.2	26	40
***Heterokaryon incompatibility***	1/11	9.1	1	0
***Ferroptosis***	10/68	14.7	6	4
***NLR***	6/45	13.3	1	5
***Apoptosis***	1/32	3.1	1	0
**Carbohydrates**				
**Gene ID**	**Predicted protein function**	**Gene name**	**log2FC**	***p*-value (FDR)**
An15g05370	Polygalacturonase II	*pgaII*	5.32	1.1E-02
An06g00170	α-galactosidase	*aglA*	3.23	2.2E-02
An12g05010	Acetyl xylan esterase	*axeA*	2.84	2.7E-06
An09g01190	Endo-l,5-α-L-arabinase	*abnA*	2.78	2.0E-02
An15g02300	α-arabinofuranosidase B	*abfB*	2.63	4.7E-02
An01g11520	Polygalacturonase	*pgaI*	1.68	9.5E-03
An01g00780	Secreted endo-1,4-xylanase	*xynB*	1.41	1.1E-02
An11g07660	Putative glucan exo-1,3-beta-glucosidase	*exgD*	-1.01	4.1E-02
An17g00520	β-glucosidase	*bglI*	-1.18	1.5E-02
An18g03570	β-glucosidase	*bglA*	-1.26	2.4E-02
An01g01870	Avicelase III	*eglC*	-1.42	2.6E-02
An04g09890	1,3-α-glucan synthase	*agsA*	-1.44	4.7E-02
An09g00260	α-galactosidase	*aglC*	-1.67	4.3E-02
An04g06920	Extracellular α-glucosidase	*agdA*	-1.79	2.1E-02
An12g08280	Extracellular exo-inulinase	*inuE*	-2.58	4.4E-03
An07g00350	α-glucosidase	*agdG*	-2.72	1.0E-02
An02g12450	Exo-polygalacturonase	*pgxC*	-3.07	7.0E-04
An15g07810	α-glucan synthase	*agsB*	-3.36	1.1E-02
An10g00870	Pectate lyase A	*plyA*	-3.71	1.5E-02
An11g03200	Inulase	*inuA*	-3.81	3.7E-02
An15g07760	Mannan endo-1,4-β-mannosidase	*man26A*	-4.3	5.3E-03
***Proteases***				
**Gene ID**	**Predicted protein function**	**Gene name**	**log2FC**	***p*-value (FDR)**
An01g00530	Extracellular acidic protease	*pepB*	7.4	9.2E-03
An14g04710	Aspergillopepsin A	*pepA*	6.51	9.0E-05
An08g04640	Sedolisin family protease	*protB*	4.88	2.0E-04
An15g07700	Aspergillopepsin II	*protD*	3.97	1.2E-03
An07g08030	Serine carboxypeptidase	*pepF*	3.58	4.2E-04
An16g09010	Protease	*protH*	1.97	4.5E-03
An08g04490	Secreted lysosomal Pro-Xaa carboxypeptidase	*protA*	1.95	3.5E-02
An02g03760	ATP-dependent peptidase	*pim1*	-1.02	2.2E-04
An11g01110	Ortholog(s) have role in proteolysis	*aorO*	-1.85	1.4E-02
An01g00370	Pepsin family protease	*pepAb*	-1.91	4.0E-02
An01g02070	Metalloendopeptidase	*mep*	-2.18	4.2E-02
***Lipases***				
**Gene ID**	**Predicted protein function**	**Gene name**	**log2FC**	***p*-value (FDR)**
An09g02180	Triacylglycerol lipase	N/A	5.14	9.7E-03
An16g01880	Lysophospholipase	*lipanl*	3.76	9.8E-04
An09g02270	Triacylglycerol lipase	N/A	3.07	8.5E-03
An16g08870	Triacylglycerol lipase	N/A	2.51	1.6E-03
An03g06560	Triacylglycerol lipase	N/A	2.5	1.5E-05
An18g06580	Triacylglycerol lipase	N/A	1.67	1.1E-02
***Cell wall remodelling***				
**Gene ID**	**Predicted protein function**	**Gene name**	**log2FC**	***p*-value (FDR)**
An12g05330	ClassV Chitinase	*cfcK*	2.59	7.2E-03
An02g02340	Chitin synthase	*chsL*	-1.01	2.9E-05
An02g02360	Chitin synthase	*chsM*	-0.93	2.9E-05
An11g07660	Exo-β-1,3-glucanase	*eglB*	-1.01	4.1E-02
An02g06830	MAPKKK with function in CWI signalling	*bckA*	-1.21	5.5E-03
An08g09030	Chitinase	*cfcB*	-1.38	9.5E-03
An04g09890	α-1,3-glucan synthase	*agsA*	-1.44	4.7E-02
An18g03740	MAPKK with function in CWI signalling	*mkkA*	-1.48	8.5E-06
An02g03980	Transglycosidase required for β-1,6 glucan biosynthesis	*kslA*	-1.67	2.9E-03
An06g00360	GPI-anchored endo-mannanase	*dfgF*	-2.09	1.5E-04
An09g00670	GPI-anchored 1,3-β-glucanosyltransferase	*gelD*	-2.43	5.3E-03
An03g06220	GPI-anchored 1,3-β-glucanosyltransferase	*gelE*	-2.55	4.2E-03
An08g05290	Chitin synthase	*chsG*	-2.79	2.7E-02
An15g07810	α-1,3-glucan synthase	*agsB*	-3.36	1.1E-02
***Transporters***				
**Gene ID**	**Predicted protein function**	**Gene name**	**log2FC**	***p*-value (FDR)**
An01g08010	Iron ion homeostasis, vacuolar membrane	*cccA*	2.37	4.9E-02
An01g04690	ATP transport, peroxisomal membrane	*antA*	2.03	1.0E-02
An11g05350	Oligopeptide transporter	*optE*	1.97	1.1E-02
An16g02000	GABA permease	*gabA*	1.74	2.1E-02
An11g03700	Hexose transporter	*hxt1*	1.66	1.1E-02
An06g00300	UDP-Gal*f*-transporter	*ugtB*	1.58	1.1E-02
An15g03200	Thiamine transporter, mitochondrial membrane	*tpc1*	1.12	3.9E-02
An12g00870	Malate permease	*ssu1*	-1.08	4.9E-02
An12g07450	Hig-affinity sugar/H+ symporter	*mstA*	-1.25	1.2E-02
An15g07460	Oligopeptide transporter	*optG*	-1.58	3.2E-02
An08g05450	ATPase	*mdr4*	-1.63	3.4E-02
An08g03850	Quinate permease	*qutD*	-1.81	4.8E-07
An12g04180	Aspartate transporter	*agtA*	-1.99	1.1E-02
An15g07190	Zinc transporter, endoplasmic reticulum membrane	*zrfA*	-2.25	6.5E-04
An02g03540	Hig-affinity hexose/H+ symporter	*mstE*	-2.28	7.2E-05
An02g14410	Ammonium transporter	*meaA*	-2.9	4.1E-02
An08g05670	Nitrate transporter	*crnA*	-3.88	6.8E-03
An07g06240	MFS transporter, ferric triacetylfusarinine C transport	*mirD*	-5.28	1.8E-02
An08g03200	Ammonium transporter	*mepA*	-5.73	8.8E-03
***Transcription factors***				
See Table 4				
***Heterokaryon incompatibility***				
**Gene ID**	**Predicted protein function**	**Gene name**	**log2FC**	***p*-value (FDR)**
An15g06140	Ortholog of *A. nidulans tinC* (AN9067) and *hetC* (AN2167)	N/A	1.11	4.8E-02
***Ferroptosis***				
**Gene ID**	**Predicted protein function**	**Gene name**	**log2FC**	***p*-value (FDR)**
An16g04760	Aldehyde reductase	N/A	2.98	1.1E-02
An04g00740	Sterol carrier	N/A	2.77	1.1E-02
An01g04830	Myb-like DNA-binding protein	*flbD*	2.34	2.0E-03
An02g12430	Isocitrate dehydrogenase	*icdA*	1.27	9.0E-03
An02g12260	γ-glutamylcyclotransferase activity	N/A	1.23	6.0E-03
An02g08110	Glutathione peroxidase	*gpxA*	1.09	6.0E-03
An04g05060	Ortholog(s) have role in cellular potassium ion homeostasis	*aslA*	-1.04	2.0E-03
An13g00890	Protein kinase	N/A	-1.08	1.3E-02
An18g04410	Cysteine dioxygenase	N/A	-1.55	5.0E-02
An04g03360	Peroxiredoxin	*prx1*	-3.31	1.0E-03
***NLR***				
**Gene ID**	**Predicted protein function**	**Gene name**	**log2FC**	***p*-value (FDR)**
An18g00540	Role in nucleoside metabolism	N/A	-2.36	4.0E-03
An08g11190	Ankyrin	*ank1*	-2.58	4.3E-02
***Apoptosis***				
**Gene ID**	**Predicted protein function**	**Gene name**	**log2FC**	***p*-value (FDR)**
An16g00710	Calcium ion binding	N/A	2.46	4.0E-03

Gene IDs and predicted gene functions after Pel et al. (2007) and FungiDB (Basenko et al., 2018). For complete gene list, see Additional File 4. The *p*-value [*p*-value (FDR)] has been corrected for false discovery rate after Benjamini and Hochberg (1995). The table shows a quantitative analysis of differentially expressed genes (top) and selected genes of the function categories (below).

Notably, carbohydrate lyases and proteases were both up- or downregulated, while most lipases were upregulated only (Table 3). Interestingly, genes active in cell wall remodeling such as chitin and glucan synthases as well as chitinases were mostly downregulated, in agreement with downregulation of genes whose protein products are known as regulators of the cell wall integrity pathway (e.g., MkkA, BckA). The complete list of differentially expressed genes is summarized in Additional Files 2, 4, whereas Table 3 highlights some selected genes.

To understand, which regulators might drive these global changes, we searched among differentially expressed genes with an FDR adjusted *p*-value below 0.05 but with no log2 fold change restriction. This enlarged the differentially expressed gene set to 1,501 genes (Additional File 2). Previous studies reported that even minor changes in transcription factor gene expression (10–15%) can translate into large gene expression responses of individual target genes (Naqvi et al., 2023). The lowest fold change observed among transcription factors with an FDR adjusted *p*-value <0.05 was 0.63 which corresponds to a 55% change in expression. With this criterium, 91 out of 691 predicted transcription factor genes in *A. niger* (13%) were differentially expressed. Table 4 highlights selected transcription factors and other regulators for which a function has been verified in Aspergilli. Interestingly, transcription factor genes that responded to induced expression of *anafp* have a predicted function in asexual development (*flbC*, *flbD*, *sfgA*, *ace2*), DNA damage response (*chkc, kusA, kueA*), general stress response (*msnA*), fatty acid catabolism (*farA*), and calcium-, potassium- and iron ion homeostasis (*aslA, sreA*), respectively. One transcription factor which also responded to *anafp* expression is *yap1*, with mammalian orthologs important for apoptosis. Notably, also cell wall integrity regulators were downregulated (such as *bckA, mkkA*) including some of their effector genes (*agsA/B*, *acuH*; Table 2; Additional Files 2, 4).

**Table 4:** Genes encoding regulators that were differentially expressed upon *anafp* overexpression in *A. niger*.

**Gene ID**	Gene name or symbol	**Product description**	log2FC	***p*-value (FDR)**
An02g05420	*flbC*	Putative C2H2 transcription factor, conidium formation	2.39	8.03E-03
An01g04830	*flbD*	Myb-like DNA-binding protein; conidium formation	2.34	1.68E-03
An01g02370	*sreA*	DNA binding activity and role in cellular iron ion homeostasis	-0.75	7.11E-03
An14g00920	*farA*	Fatty acid-related transcription factor	-0.81	2.08E-02
An11g06950	*nimO*	Protein serine/threonine kinase activator activity	-0.83	1.28E-02
An15g03280	*chkC*	DNA damage checkpoint and nucleus localization	-0.83	4.73E-02
An02g14490	*acuK*	Gluconeogenesis	-0.87	3.01E-02
An11g07980	*yap1*	Apoptotic process, asexual sporulation, asperthecin & emericellin biosynthetic process	-0.92	4.87E-03
An02g09780	*sfgA*	Conidium formation	-0.93	1.71E-03
An15g02700	*kusA*	Ku70 ortholog involved in non-homologous end-joining	-1.00	1.39E-02
An04g05060	*aslA*	Cellular potassium ion homeostasis	-1.04	2.22E-03
An07g05980	*kueA*	Ortholog of Ku80	-1.12	2.49E-02
An04g08600	*araR*	L-arabinose responsive transcriptional activator	-1.13	3.05E-04
An04g03980	*msnA*	Cellular response to calcium ion, heat, oxidative stress	-1.47	1.61E-05
An02g07000	*ace2*	Asexual spore wall assembly	-2.35	1.27E-06

Gene IDs and predicted gene functions after Pel et al. (2007) and FungiDB (Basenko et al., 2018). The *p*-value has been corrected for false discovery rate after Benjamini and Hochberg (1995).

Taken together, the differentially expressed gene set of this study confirms our previous comparative transcriptomic analyses and strongly points toward a connection between AnAFP, nutrient mobilization & transport, and asexual development in *A. niger* under carbon starvation conditions. It appears that AnAFP specifically stimulates upstream regulators of conidiophore development such as *sfgA*, *flbC*, and *flbD*, while expression of the regulatory genes executing sporulation such as *brlA, wetA*, and *abaA* remain unaffected. This is in excellent agreement with data from Figure 3 that demonstrated that sporulation is not affected by the presence of AnAFP in STS4.10.

## 4 Discussion

AnAFP belongs to the antimicrobial peptide family with members across almost all kingdoms of live (Feurstein et al., 2022) and has thus withstood millions of years of evolutionary selection pressure. Some antimicrobial peptides have been reported to have acquired secondary functions next to their primary antimicrobial activity against nutrient competitors (Meyer and Jung, 2018; Yeaman and Yount, 2007; Yount and Yeaman, 2006). Examples include the ability of cathelicidins to contribute to wound healing in human cells (Lee et al., 2018; Arnett and Seveau, 2011), the function of hepcidin in human iron metabolism (Patel and Akhtar, 2017), the involvement of gloverins in insect development (Xu et al., 2012; Hwang and Kim, 2011), and the chitin binding and wound healing activity of AMPs in shrimps and horseshoe crabs (Otero-Gonzáiez et al., 2010).

**Figure 7:**
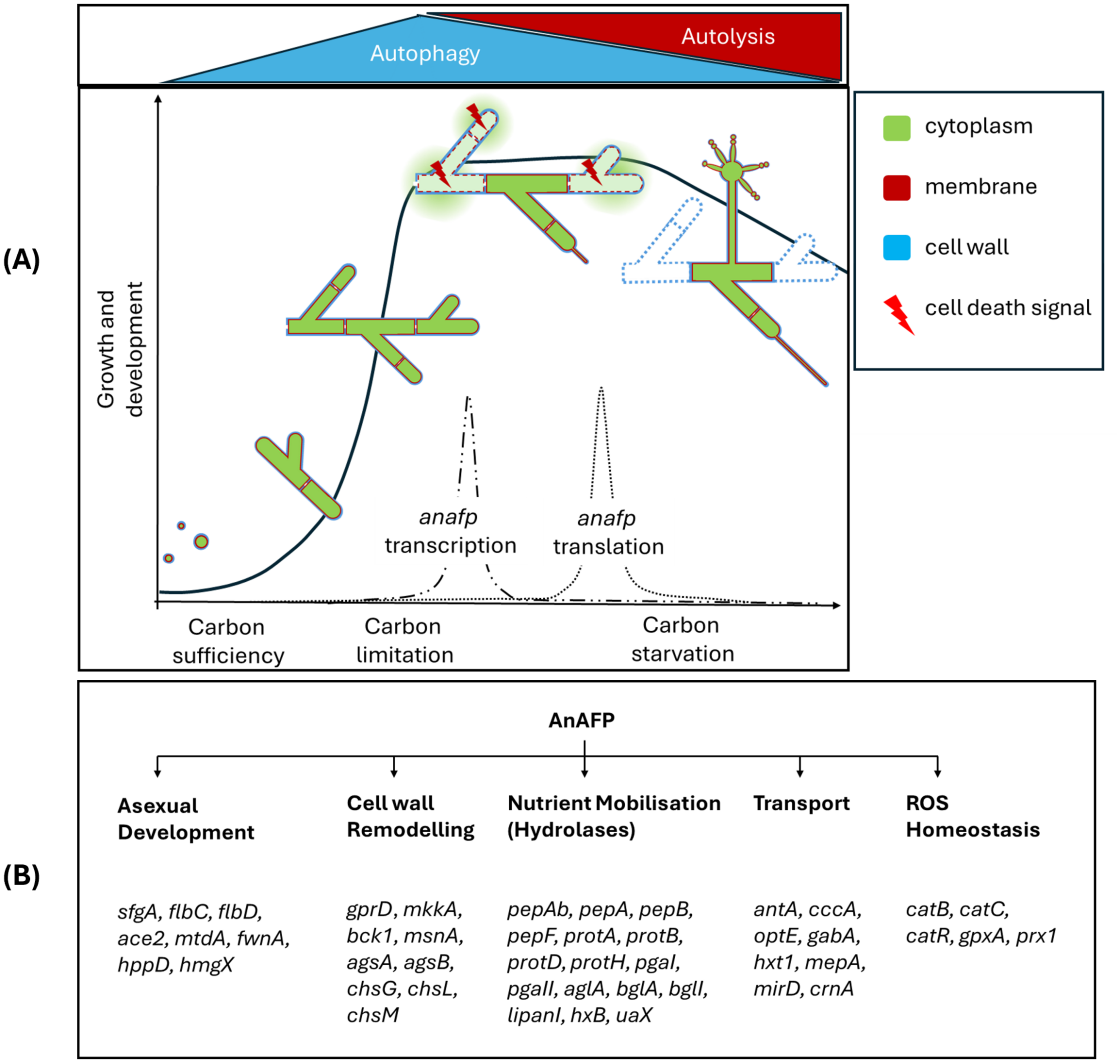
Working model for the intracellular function of AnAFP in *A. niger* based on data from this work and previous studies (Paege et al., 2016; Nitsche et al., 2012). (A) Depicted is the asexual life cycle of *A. niger* cells under solid-state and submerged growth conditions including spore germination, hyphal growth, vegetative mycelium formation and outgrowth of conidiophores. During exponential growth phase, hyphal compartments start to form visible vacuoles to recycle misfolded proteins. When nutrients in the environment become limiting, post-exponential growth starts and vacuolar size increases to provide nutrients and energy through autophagy. First conidiophores start to appear. During this narrow time window, the *anafp* gene becomes transcribed with a peak-like mRNA expression profile (Paege et al., 2016). Time-delayed (dependent on the cultivation condition), *anafp* mRNA becomes translated and intracellularly AnAFP is formed to support autophagic process. Throughout prolonged nutrient starvation, however, more nutrients need to become recycled to fuel conidiophore development. Hyphal compartments with higher AnAFP presence thus occur. This supports autolysis to become dominant over autophagy, i.e., some hyphal compartments with high amounts of AnAFP decrease their vacuole size, disintegrate their cell walls, so that eventually the cytoplasm disappears and such hyphal compartment significantly reduces its diameter. Finally, the vegetative mycelium population enters the death phase and autolysis of individual hyphal compartments provides further nutrients to support conidiophore formation and eventually sporulation. Altogether, this sequence ensures survival of the species. (B) AnAFP is embedded in many cellular regulatory processes that drive these different developmental stages. Selected genes are indicated that become differentially expressed as response to *anafp* overexpression. See Discussion for details.

We propose here a working model in which AnAFP also exerts a function for *A. niger* that goes far beyond its antifungal activity against nutrient competitors (Figure 7). Transcription of the *anafp* gene peaks in vegetative hyphae, conidiophores as well as hyphae in their vicinity but neither in the conidiophore vesicle nor in conidiospores (Figures 1, 5; Supplementary Video S1), demonstrating that transcription of the *anafp* gene is under tight spatial and temporal control. In general, conidiophore formation in Aspergilli follows a differentiation program which becomes initiated during carbon or nitrogen deprivation and is accompanied by a severe growth cessation of vegetative hyphae (Adams et al., 1998). Thus, *anafp* transcription is limited to vegetative hyphae competent for conidiophore formation, i.e., when their growth becomes repressed to allow differentiation into conidiophores. Notably, translated AnAFP provokes a sudden reduction in hyphal diameter of those hyphal compartments where it becomes expressed (Figure 5). From these data we propose that AnAFP is important to support the asexual differentiation program, i.e., asexual sporulation, in *A. niger* during nutrient, especially carbon limitation. We predict that AnAFP drives nutrient mobilization through autophagic recycling of vegetative hyphae to fuel growth of neighbouring compartments and conidiophores, and thus survival of the whole colony during phases of starvation (Figure 7).

We also report here that expression of the *anafp* gene is regulated at the post-transcriptional level. Although the *anafp* mRNA was produced in high amounts under the control of the Tet-on promotor, translation only occurred in the presence of its native *anafp* 5’ and 3’ UTRs (Figure 2). In agreement, the *anafp::egfp* fusion showed intracellular green fluorescence only after prolonged nutrient starvation despite being controlled under the Dox-induced Tet-on system and carrying its 5’ and 3’ UTRs (Figure 5). This data perfectly match our previous observations (Meyer and Jung, 2018) that expression of the *anafp* gene in wild type *A. niger* peaked 16 h after carbon starvation while extracellular AnAFP protein was detected only 160 h post starvation (van Munster et al., 2015). Note that such discrepancy between high level mRNA and lack of extracellular protein was also observed for two other members of the AFP family, namely the PAFB protein from *P. chrysogenum* (Huber et al., 2018) and the AfpB protein from *P. digitatum* (Garrigues et al., 2016). Here, the importance of native promotor and terminator sequence for AFP expression was also reported for the AfpA protein from *P. expansum* (Gandía et al., 2022; Sonderegger et al., 2016). Note that the choice of an optimal 5’ UTR has been demonstrated to increase the production of non-antifungal peptides, e.g., the reporter protein *β*-glucuronidase in *A. oryzae* (Koda et al., 2004).

Moreover, the regulation of AnAFP translation could be influenced by the cellular location of *anafp* mRNA. This impact on translation efficiency has been reported for cells displaying polarized growth such as filamentous fungi or neuronal cells (Das et al., 2021; Staples et al., 2023). It is thought that mRNAs travel within stress granules (a phase-separated accumulation of RNAs and ribosomes) within specific regions in the cell (i.e., tip, periphery, septa), while translation is repressed until the destination is reached or a certain environmental condition is encountered. Whether this is the case for the *anafp* mRNA in *A. niger* remains to be shown.

We therefore conclude that expression of the *anafp* gene in *A. niger* is under sophisticated transcriptional and post-transcriptional control and encodes a protein that exerts its function first intracellularly and then extracellularly. We furthermore propose that AnAFP exerts two different intracellular functions during growth of *A. niger* as summarized in Figure 7: (i) During early starvation (here 4 h after carbon depletion), it can induce the (premature) onset of autophagy to translocate cytoplasmatic proteins (here eGFP) into vacuoles in an Atg1 and Atg8 dependent manner for nutrient recycling (Figure 6). (ii) During prolonged starvation (here 30 h after carbon depletion), it assists in cellular lysis of cell compartments where it is expressed to fuel neighboring cell compartments and conidiophores with nutrients. This activity results in the sudden reduction of the hyphal diameter (Figure 5). Our transcriptomics analyses (Tables 3, 4) reinforce the autolytic role of AnAFP as hundreds of genes important for nutrient mobilization and metabolism become differentially expressed upon *anafp* overexpression. This also includes genes with a putative function in lytic cell death forms such as ferroptosis, heterokaryon incompatibility and NLR-associated cell death (Additional File 4).

We have previously analyzed the transcriptomic response of *A. niger* during carbon starvation in bioreactor cultures and could uncover that the mycelium responds with autophagy during early phases of nutrient deprivation but with autolysis during later stages of starvation (Nitsche et al., 2012). In parallel to the differential expression of autolytic genes, AnAFP also induced the expression of genes which respond to the presence of reactive oxygen species (ROS), as reflected by the enrichment of GO terms detoxification (GO:0098754) and hydrogen peroxide metabolic process (GO:0042743) (Additional File 3). Interestingly, an intertwined activity of antimicrobial peptides, reactive oxygen species (ROS) and compartmentalized cell death processes is a phenomenon described for the innate immune system of plants and animals and was most recently also proposed to exist in fungi (Gaspar and Pawlowska, 2022). ROS, a key characteristic of fungal apoptotic-like cell death is also often used as a way to mobilize nutrients (Gaspar and Pawlowska, 2022). Given the amphipathic nature of antimicrobial peptides and their involvement in immune responses across eukaryotic kingdoms, antifungal peptides could have evolved two functions: one to fight fungal nutrient competitors and one to act as cannibal-toxin to ensure species survival under nutrient scarcity. Regardless of the form of cell death which may be triggered by AnAFP, we argue that the liberated nutrients are used by hyphal compartments in the vicinity for survival and the establishment of conidiophores. The latter would explain why AnAFP::eGFP signatures can be found in conidiophores (Figure 5). Remarkably, a similar global transcriptional response was most recently published for AfpB in *P. digitatum* (Bugeda et al., 2020; Ropero-Pérez et al., 2023). We thus performed a comparative GO term analysis of differentially expressed genes in both strains *A. niger* and *P. digitatum* upon increased AnAFP/AfpB expression which uncovered an enrichment in hydrolase and transmembrane transporter expression (Additional File 5). Likewise, transcriptome data for PeAfpA from *P. expansum* also report significant changes in the expression of genes involved in cell wall remodeling, carbohydrate lysis, and nutrient transport (Ropero-Pérez et al., 2024), altogether suggesting that the observed role of AnAFP in nutrient mobilization in *A. niger* can also be extended to other AFP family members.

Interestingly, the mobilization of genes important for hydrolases, transporters and programmed cell death is a phenomenon which has also been observed in more distantly related eukaryotic cells: namely in the process of germination in rice and barley. During germination thousands of genes are differentially expressed through the activity of specific transcription factors, one of which is a Myb-like DNA binding protein named MYBGA (Hong et al., 2012). The ortholog in Aspergilli is FlbD, a transcription factor with central role in asexual development in *A. niger* (Krijgsheld et al., 2013) and in (a) sexual differentiation and apoptosis in *A. nidulans* (Krijgsheld et al., 2013; Arratia-Quijada et al., 2012). Table 4 highlights that the *flbD* gene and its target gene, the *flbC* gene encoding the transcriptional activator FlbC [which itself activates expression of the *brlA* gene (Kwon et al., 2010)] are both upregulated upon *anafp* overexpression. In agreement, the *sfgA* gene encoding the repressor of both FlbD and FlbC is repressed. These genes play a crucial role in Aspergilli for the regulation of conidiophore and conidiospore formation, overall governed by the FluG-BrlA cascade (Park and Yu, 2012). Note that deletion of one of the key developmental regulators FlbA upstream of FlbD induces an autolysing phenotype in *A. niger* and increases *anafp* mRNA levels fivefold (Paege et al., 2016). On the other hand, *anafp* expression is independent of BrlA (Paege et al., 2016), which is the transcription factor that drives progression of the asexual developmental cascade toward sporulation, i.e., BrlA is key for the formation of phialides and conidia together with the transcription factors AbaA and WetA (Ojeda-López et al., 2018). In *A. nidulans*, it has been demonstrated that parts of this cascade not only regulate asexual reproduction but can also lead to the induction of autolysis through the production of proteinases and chitinases (Tamás et al., 2005). Similarly, the transcription factor FlbC was shown to play a role in the mobilization of hydrolase genes in *A. oryzae* (Tanaka and Gomi, 2021).

There is increasing evidence that the FluG-BrlA cascade has functions in Ascomycota (*Pezizomycotina*) that can go beyond asexual development and show their involvement in nutrient mobilisation through regulation of cell wall integrity and cell lysis (Ojeda-López et al., 2018; Guo et al., 2022). As discussed, our data show a strong differential regulation of genes involved in nutrient mobilization through autolysis and ROS.

Interestingly, genes active in autolysis have recently been reported to also contribute to conidiophore development and sporulation in *A. fumigatus* through the cell wall integrity pathway (CWI), whereby its central transcriptional factor RlmA directly regulates expression of the *flbB*, *flbC*, *brlA*, *abaA* genes (Rocha et al., 2020; Binder et al., 2011). Congruently, members of the CWI pathway in *A. niger* are consistently downregulated in response to *anafp* upregulation (Table 2). The capability of *A. niger* to detect and respond to cell wall stress is, however, not only dependent on the CWI pathway (transcription factor RlmA), but also on the general stress response pathway (transcription factor MsnA) and the calcium-calcineurin pathway (transcription factor CrzA) (Fiedler et al., 2014). All three transcription factors have also been shown to be important for asexual development in other Aspergilli (Rocha et al., 2020; Chang et al., 2011; Ren et al., 2021; Kovács et al., 2013). It is thus conceivable that *anafp* induced downregulation of the CWI pathway in *A. niger* reflects cell wall weakening, whereby depolymerized carbohydrates again serve nutrient mobilization for the generation of conidiophores.

## 5 Conclusion

This work uncovered that the antifungal peptide AnAFP, which was so far described to act exogenously as a secreted protein, exerts an important intracellular function for *A. niger*. We propose that the protein functions as a cannibal toxin that fine-tunes timing and efficiency of self-digestion during carbon starvation. We also conclude that AnAFP does not act as an ON/OFF switch for autophagy or autolysis as its deletion is neither lethal nor its down- or upregulation affects the general ability of *A. niger* to undergo asexual development and sporulation. We further show here that its expression is regulated in a very sophisticated manner in time and space at both the transcriptional and translational level. We propose that this ensures that only hyphal compartments competent for asexual development undergo autophagy and autolysis. Data from transcriptome analyses suggest that *anafp* expression is therefore embedded in many transcriptional regulatory programs which eventually ensure that the diameter of competent compartments become reduced to fuel neighbouring compartments and conidiophores. The intertwining of all subcellular and regulatory processes needs to be discovered in the future and – because of the entanglement of dying and outgrowing cells – will require new spatial and single cell omics approaches for fundamental understanding.

## Data Availability

The datasets presented in this study can be found in online repositories. The names of the repository/repositories and accession number(s) can be found in the article/Supplementary material.
