# Supplementary material for: The antifungal peptide AnAFP from Aspergillus niger promotes nutrient mobilization through autophagic recycling during asexual development
Source: Front Microbiol. 2025 Jan 24;15:1490293. doi: 10.3389/fmicb.2024.1490293 (PMC11802824; doi:10.3389/fmicb.2024.1490293)
Supplement: Supplementary file 3 [file Data_Sheet_1.zip › Additional File 6 ( Code for image processing ).pdf]

Additional File 6: Code used for image processing of z-stacks in nuclear chromatin condensation assay

```
macro "Process and measure Fluorescence images (grey-value) [g]" {
//Important: The File>Open command will open multiple file if you enable “Use
jFileChooser to open/save” in Edit/Options/Input-Output. Needs to be enabled, is
disabled by default!
print("Start Processing");
setBatchMode(true);
import=getDirectory("Choose >>sample<< input directory");
for (i=0; i<10; i++) {
stack=i+1;
open(import+"/CFP/"+stack);
name=getTitle();
name=replace(name, "/CFP/", "");
rename(name);
print("Fluorescence loading "+"Stack_"+stack+" complete");
imageTitle=name;
input=import;
output=input+"/output/";
outputCFP=input+"/outputCFP/";
outputControl=input+"/outputControl/";
outputComposites=input+"/outputComposites/";
File.makeDirectory(output);
File.makeDirectory(outputCFP);
File.makeDirectory(outputControl);
File.makeDirectory(outputComposites);
print("Creating subfolders "+"of Stack_"+stack+" complete");
run("Z Project...", "projection=[Max Intensity]");
selectWindow(imageTitle);
run("Z Project...", "projection=Median");
nameMaxStack="MAX_"+imageTitle;
nameMedStack="MED_"+imageTitle;
imageCalculator("Subtract create", nameMaxStack,nameMedStack);
selectWindow("Result of "+nameMaxStack);
run("Duplicate...", " ");
run("Gaussian Blur...", "sigma=10");
imageCalculator("Subtract create", "Result of "+nameMaxStack,"Result of
"+nameMaxStack+"-1");
selectWindow("Result of Result of "+nameMaxStack);
run("8-bit");
run("Enhance Contrast...", "saturated=0.5");
run("Apply LUT");
run("Auto Threshold", "method=Moments white");
run("Set Measurements...", "area mean min shape display
redirect="+nameMaxStack+" decimal=3");
run("8-bit");
run("Analyze Particles...", "size=112-1200 pixel circularity=0.30-1.00 show=Outlines
display exclude include in_situ");
```

```
imageCalculator("Add create", "Result of Result of
"+nameMaxStack,nameMaxStack);
print("Analysis "+"of Stack_"+stack+" complete");
selectWindow("Result of Result of "+nameMaxStack);
close();
selectWindow(imageTitle);
close();
selectWindow(nameMedStack);
close();
selectWindow("Result of "+nameMaxStack);
close();
selectWindow("Result of "+nameMaxStack+"-1");
close();
selectWindow("Result of Result of Result of "+nameMaxStack);
saveAs("png",outputControl+"Control_outlines_CFP_"+imageTitle);
close();
print("Saving Control_outlines "+"of Stack_"+stack+" complete");
selectWindow(nameMaxStack);
run("8-bit");
run("Cyan");
run("Apply LUT");
saveAs("png",outputCFP+"MAX_Stack_CFP_"+imageTitle);
print("Saving "+"of Stack_"+stack+" Max_Stack complete");
//Process and make composite of DIC and Fluorescence images
import2=input+"/DIC/"+stack+"/Focused/";
print("DIC image "+"of Stack_"+stack+" is\n\t"+import2);
open(import2);
imageTitleDIC=getTitle();
run("8-bit");
run("Smooth");
run("Size...", "width=1392 height=1040 depth=1 average interpolation=Bilinear");
selectWindow("MAX_Stack_CFP_"+imageTitle+".png");
imageTitleCFP=getTitle();
run("Merge Channels...", "c4="+imageTitleDIC+" c5="+imageTitleCFP+" create");
print("Processing Composite "+"of Stack_"+stack+" complete");
selectWindow("Composite");
rename("Composite_"+imageTitle);
saveAs("png",outputComposites+"Composite"+imageTitle);
close();
print("Saving Composite "+"of Stack_"+stack+" complete");
print("Stack "+stack+" complete!\n");
}
saveAs("Results", output+"Results.csv");
selectWindow("Log");
saveAs("Text", output+"Log.txt");
print("Results "+"of Stack_"+stack+" is saved
under\n\t"+output+"Results_"+imageTitle+".csv");
print("Saving Results "+"of Stack_"+stack+" complete");
print("\nMacro completed successfully!");
setBatchMode("exit & display");
```

```
}
```
