# Supplementary material for: The antifungal peptide AnAFP from Aspergillus niger promotes nutrient mobilization through autophagic recycling during asexual development
Source: Front Microbiol. 2025 Jan 24;15:1490293. doi: 10.3389/fmicb.2024.1490293 (PMC11802824; doi:10.3389/fmicb.2024.1490293)

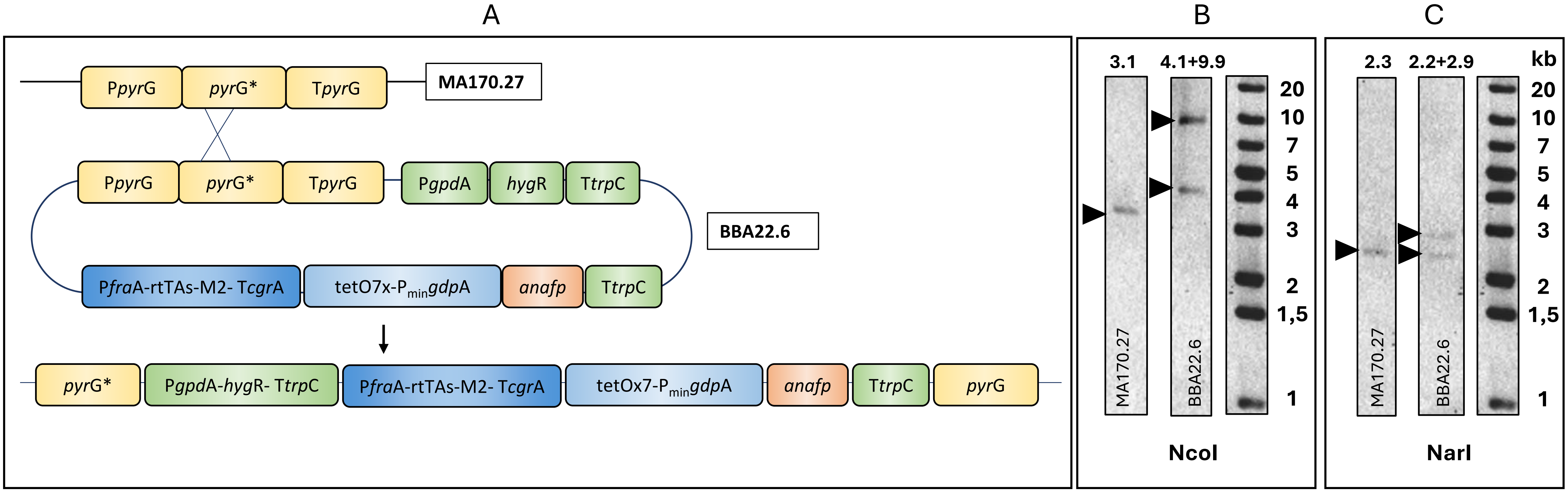

Southern Probe:(binds *pyrG*)
CCGTGATCAAAACCCACATCGATATCCTCTCTGATTTCAGCAACGAGACCATTGAGGGACTTAAGGCTCTCGCGCAGAAGCACAACTTTCTCATCTTCGAGGACCGCAAGTTCATTGACATCGGCAACACGGTCCAGAAGCAATACCACGGCGGTACCCTCCGTATCTCGGAATGGGCCCACATCATCAACTGCAGCATTCTCCCTGGTGA
GGGTATCGTCGAGGCTCTCGCTCAGACGGCGTCTGCACCGGACTTCGCCTACGGCCCCGAACGCGGTCTGTTGATCTTGGCAGAGATGACCTCTAAGGGCTCCTTGGCTACCGGCCAGTACACTACTTCCTCGGTCGATTATGCCCGGAAATACAAGAACTTCGTTATGGGATTCGTGTCGACGCGCGCGTTGGGTGAGGTGCAGTCGG
AAGTCAGCTCTCCTTCGGATGAGGAGGACTTTGTGGTCTTCACGACTGGTGTGAACATTTCTTCCAAGGGAGATAAGCTTGGTCAGCAGTACCAGACGCCCGGATCGGCTATCGGCCGGGGTGCTGACTTCATTATCGCGGGTCGCGGTATCTACGCCGCGCCGGATCCGGTGCAGGCTGC

Figure S1: Cloning strategy and southern blot verification of *A. niger* BBA22-6 (Tet-On-*anafp*, no UTR). (A) Schematic of the *A. niger* MA170.27 (Δ*anafp*) (progenitor) *pyrG* locus (*dysfunctional) [1], corresponding donor DNA (plasmid DNA) integrating via single recombination and resulting *pyrG* locus in BBA22.6 (B, C) Southern blot data of BBA22.6 and MA170.27 when cutting with EcoR1 and Bgl1. Expected band sizes are marked with a black arrowhead and are written above the blot lane in kbp. (D) Southern probe sequence

[1] Arentshorst, Mark, Ellen L. Lagendijk, and Arthur FJ Ram. "A new vector for efficient gene targeting to the *pyrG* locus in *Aspergillus niger*." *Fungal Biology and Biotechnology* 2 (2015): 1-5.

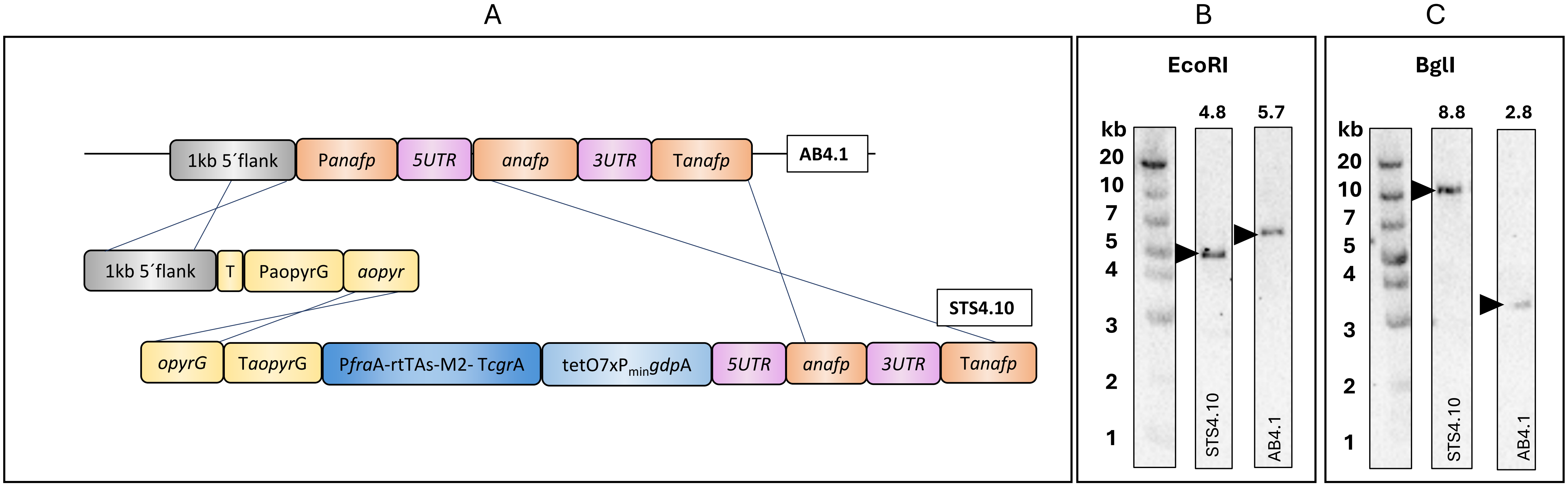

D

Southern Probe:(binds *anafp* terminator)

AGCTGTACAAGTAATTGGGTTGATTCGGTTTCACAGGGACATAGGCCAATTTTTGGCTGTTTCGGTGTTTCTCCAGCTTGCCATAGCATGAGATTATTTCGTGTCTTTCAACAATGCCCGATTATCTTGTGTCATGGTGGGATGGCGGTGAGTGAGCTATGGATTTTGTCCGTTGCTGGGGTTGAATATGTTAGTTGTTGTTTGCAGTTCTTGT
TATCATCGCGCTATATTGGTATCTAGCTAGCTCTACCTTTATCTCAGTGTTGTCTGTTGACTTGCTGTTATTATTTAAGGTCATTCTACTAGTAGCTGTATCCCTGATGGTTGTTAAGTGCGTTGGCCAGCTGGGAAGTAGTATATATTACATAGCCAATTCTGTATACTATAGAACACACGCAACTGTAGCTTTATTTACCTTTCCAATCTAGGAGCC
GCTAAAGGGATCAGGATATCTGAGAATAAAGCAATAGCGAATAGAGTCTCGTATTCATCGTCATCATTATTAGCGGAAAGACTAGCGCTGGTGAAGGGTGCTATGTACCCTGGTTCTGCTCCGTAAGCATAGACATATTTCTTCAAAAGAATATGACGACTGTACCGCCACGCTGATATGCGATGGGCCAGTGCTGCCCGCCATACTGCACAA
CACACGGACTGGTAATCACACAACATCCTTCCTATAATCTATCCGCACGTCAGAAGCCTCCCTCGCCTCCCCATCAGCATCTGCAAACGCTGTATCGCCAAACAACTCTGCAACTTCCTCCAGCGTGCTATTGGCCGTCTCAACATAGTAGAAGTGTACCAGGCCTGCAGAGAAGAGACAGAACCCAGCAATAACAACGTAGAAGTATCCGT
TGATACCTGCGAACGCATCCGGAGATACCATGCCGATGATGAAGTTGAAGATCCAGTTGCTGGTAGTAGCCAGAGCCATTCCTTTACTGCGGCCACGCAAGGGGAAGATTTCAGCTGGTAGCAGCCATGGCATTGGACCCCAGGTGAAGCCGTATAGGATGTTGCCAGAAAATGCG

Figure S2: Cloning strategy and southern blot verification of *A. niger* STS4.10 (Tet-On-*anafp*, +UTR). (A) schematic of the *A. niger* AB4.1 (progenitor) *anafp* locus and the corresponding donor DNA (counterselectable *Aspergillus oryzae* (ao) *pyrG* gene & split marker) [1,2], integrating via double recombination. T= Terminator(ao*pyr*G) (B, C) Southern blot data of STS4.10 and AB4.1 when cutting with EcoR1 and Bgl1. Expected band sizes are marked with a black arrowhead and are written above the blot lane in kbp. (D) Southern probe sequence.

[1] Arentshorst, Mark, Jing Niu, and Arthur FJ Ram. "Efficient generation of *Aspergillus niger* knock-out strains by combining NHEJ mutants and a split marker approach." *Genetic Transformation Systems in Fungi, Volume 1* (2015): 263-272.

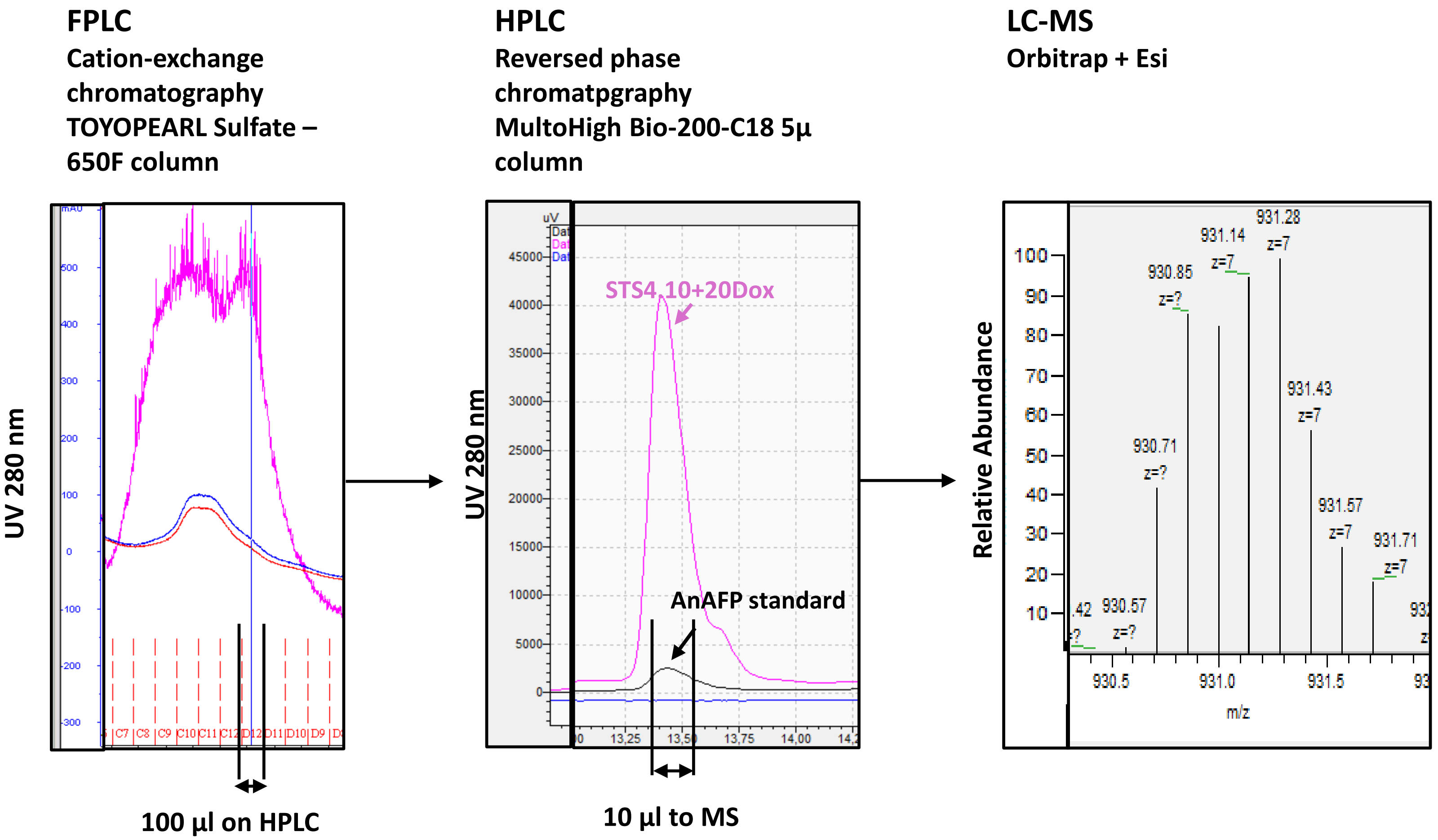

$M = m1 * z - z$

Signal	*charge*	*calculated mass (Da)*
1085.49	6	6506.96
930.57	7	6506.99
814.37	8	6506.96

Expected mass	Cys protonated	Cys deprotonated
	6512.97	**6506.92**

Figure S3: Purification of AnAFP from the Supernatant of STS4.10 (Tet-On-*anafp*, + UTR). STS4.10 was cultivated for 48 hours at 37°C in CM Media in a total volume of 50 ml in presence of 20 µg/ml Doxycycline. 20 ml of the culture supernatant was subjected to cation-exchange chromatography (FPLC). 100 µl of a 2 mL fraction was subsequently applied to reversed phase chromatography from which a 10 µl was analyzed on LC-MS. 3 prominent signals were measured in the LC-MS which correspond to the m/z value of deprotonated AnAFP with z= 6,7 and 8.

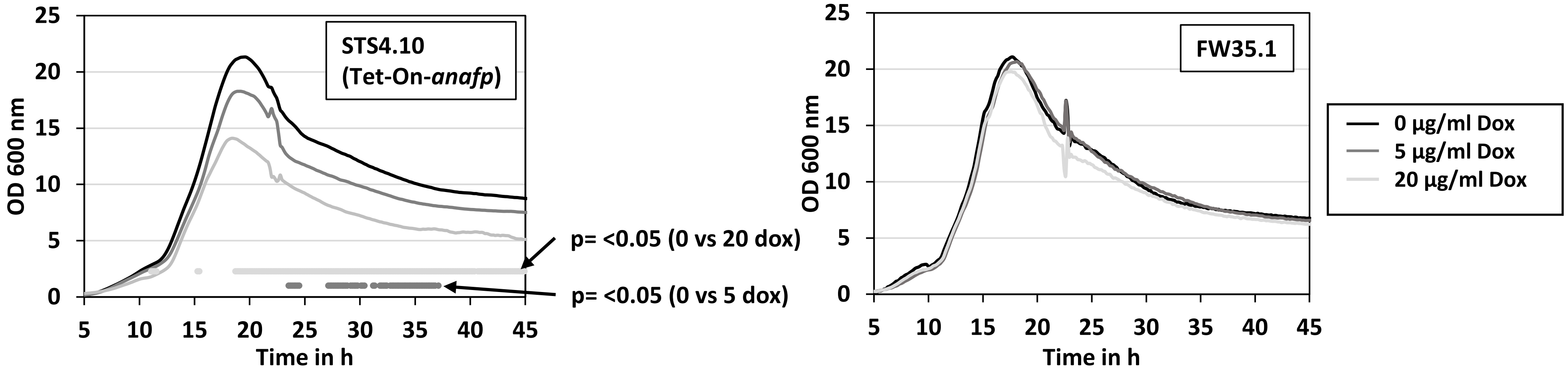

Figure S4: Optical density of a 50 ml CM shake flask culture, in presence of 0, 5, and 20 µg/ml doxycycline, cultivated over 45 hours at 30°C. Data shown include STS4.10 (Tet-On *anafp*; +UTR) and FW35.1 (progenitor; *pyrG* complemented). Each time point with a statistically significant difference (p-value < 0.05) between the doxycycline-treated and untreated samples is indicated by grey dots, with darker shading representing the comparison between 0 and 5 µg/ml doxycycline and lighter shading for 0 against 20 µg/ml. (n=3).

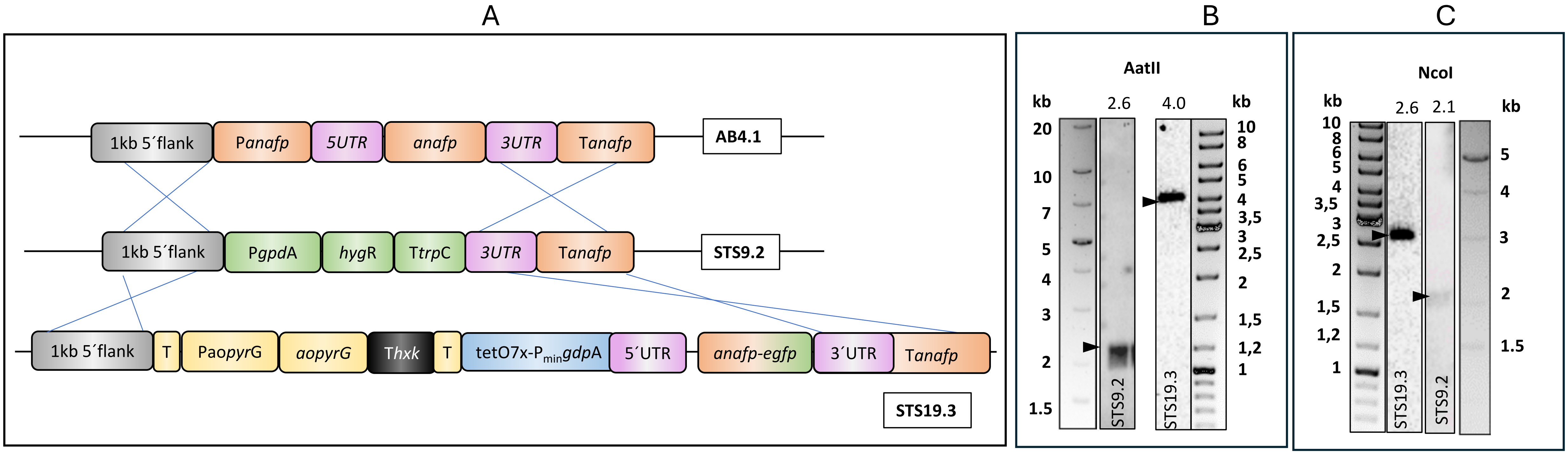

D

Southern Probe:(binds *anafp* terminator)
AGCTGTACAAGTAATTGGGTTGATTCGGTTTCACAGGGACATAGGCCAATTTTTGGCTGTTTCGGTGTTTCTCCAGCTTGCCATAGCATGAGATTATTTCGTGTCTTTCAACAATGCCCGATTATCTTGTGTCATGGTGGGATGGCGGTGAGTGAGCTATGGATTTTGTCCGTTGCTGGGGTTGAATATGTTAGTTGTTGTTTGCAGTTCTT
GTTATCATCGCGCTATATTGGTATCTAGCTAGCTCTACCTTTATCTCAGTGTTGTCTGTTGACTTGCTGTTATTATTTAAGGTCATTCTACTAGTAGCTGTATCCCTGATGGTTGTTAAGTGCGTTGGCCAGCTGGGAAGTAGTATATATTACATAGCCAATTCTGTATACTATAGAACACACGCAACTGTAGCTTTATTTACCTTTCCAATCTAGG
AGCCGCTAAAGGGATCAGGATATCTGAGAATAAAGCAATAGCGAATAGAGTCTCGTATTCATCGTCATCATTATTAGCGGAAAGACTAGCGCTGGTGAAGGGTGCTATGTACCCTGGTTCTGCTCCGTAAGCATAGACATATTTCTTCAAAAGAATATGACGACTGTACCGCCACGCTGATATGCGATGGGCCAGTGCTGCCCGCCATACT
GCACAACACACGGACTGGTAATCACACAACATCCTTCCTATAATCTATCCGCACGTCAGAAGCCTCCCTCGCCTCCCCATCAGCATCTGCAAACGCTGTATCGCCAAACAACTCTGCAACTTCCTCCAGCGTGCTATTGGCCGTCTCAACATAGTAGAAGTGTACCAGGCCTGCAGAGAAGAGACAGAACCCAGCAATAACAACGTAGA
AGTATCCGTTGATACCTGCGAACGCATCCGGAGATACCATGCCGATGATGAAGTTGAAGATCCAGTTGCTGGTAGTAGCCAGAGCCATTCCTTTACTGCGGCCACGCAAGGGGAAGATTTCAGCTGGTAGCAGCCATGGCATTGGACCCCAGGTGAAGCCGTATAGGATGTTGCCAGAAAATGCG

Figure S5: Cloning strategy and southern blot verification of *A. niger* STS19.3 (Tet-On-*anafp-egfp*, +UTR). (A) schematic of the *A. niger* AB4.1 (progenitor) *anafp* locus and the corresponding intermediate *A. niger* STS9.2 (hygromycin resistant, *pyrG* auxotroph), resulting in STS19.3 (hygromycin sensitive, *pyrG* prototroph, *pyrG* counterselectable)[1] via double recombination. T= Terminator(ao*pyr*G) (B, C) Southern blot data of STS4.10 and AB4.1 when cutting with Aat2 and NcoI. Expected band sizes are marked with a black arrowhead and are written above the blot lane in kbp. (D) Southern probe sequence.

[1]Niu, Jing, et al. "A set of isogenic auxotrophic strains for constructing multiple gene deletion mutants and parasexual crossings in Aspergillus niger." *Archives of Microbiology* 198 (2016): 861-868.

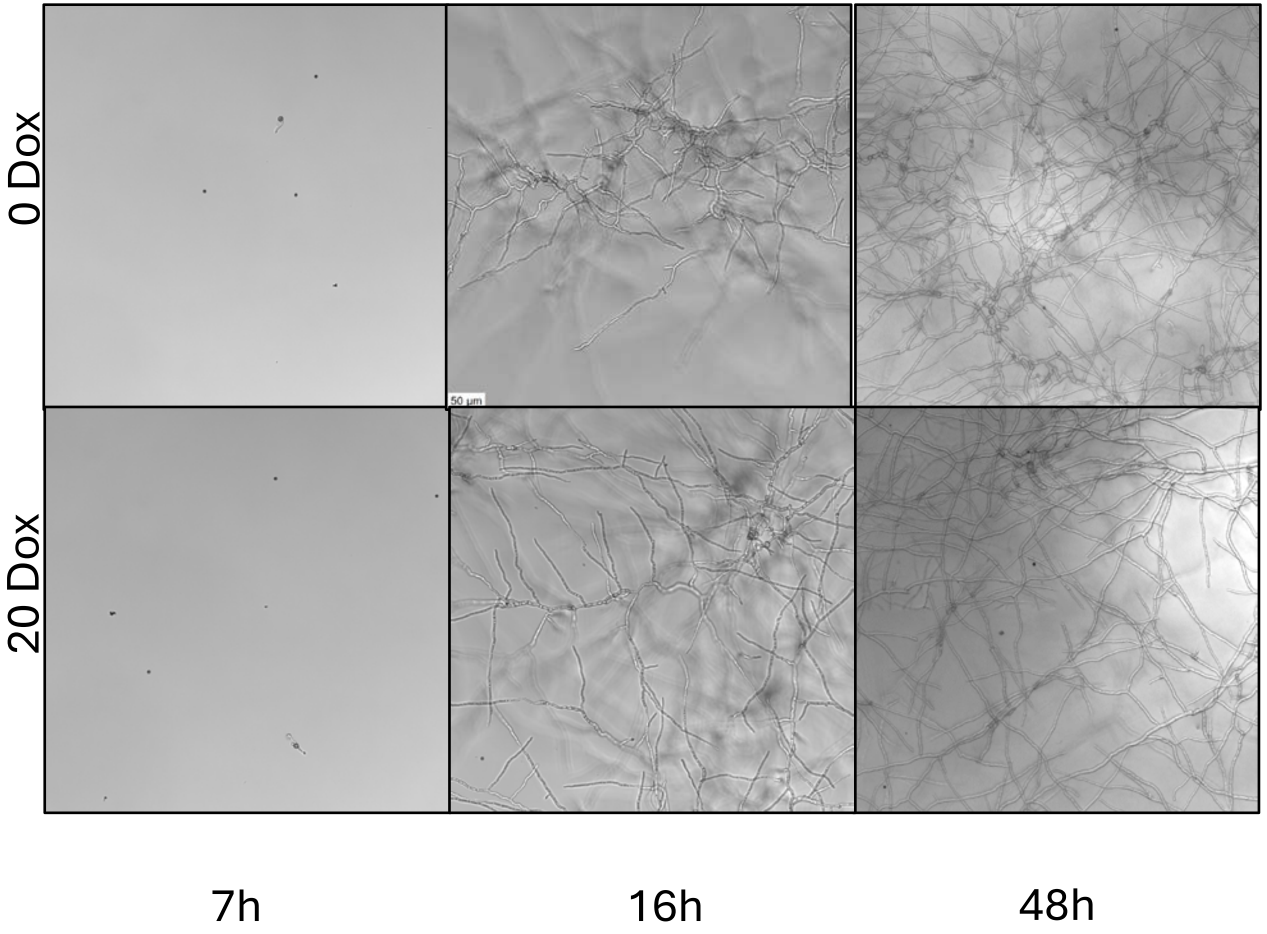

Figure S6: Growth of STS19.3 (Tet-On-*anafp::egfp*) in MM for 48 hours with no induction of starvation through media change. Dox applied at 0 h, growth at 30°C, exemplary micrographs shown after 7, 16 and 48 hours. GFP fluorescence signal was not detected (data not shown).

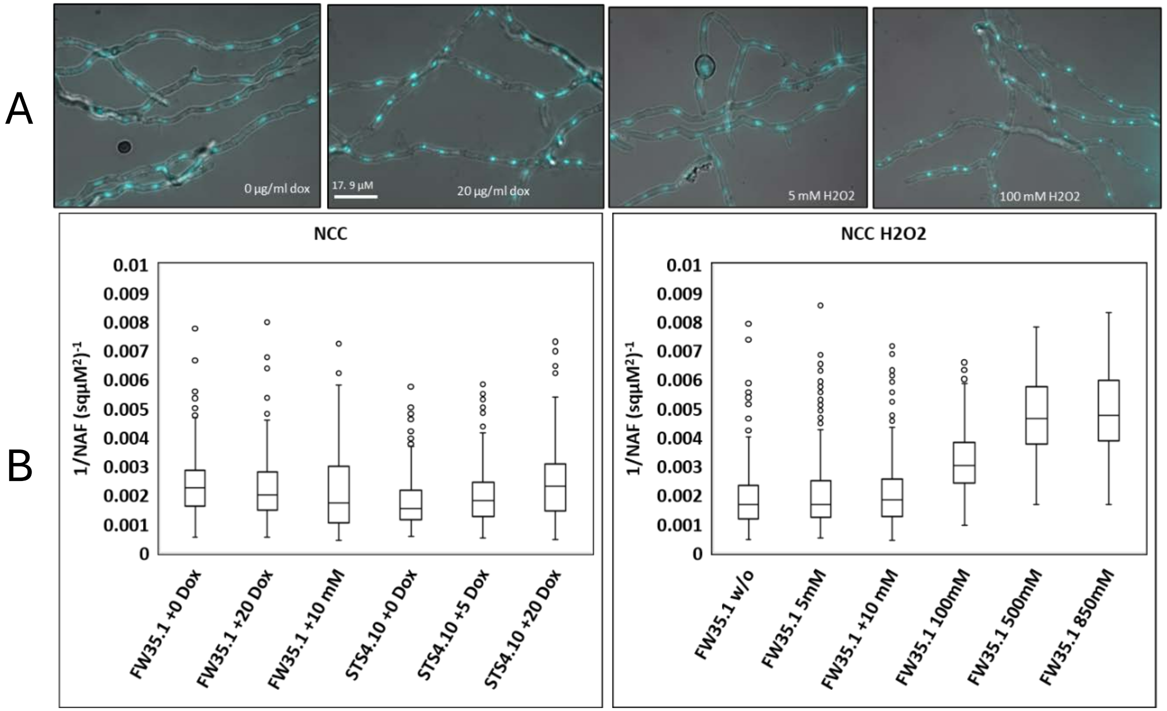

Figure S8: Nuclear chromatin condensation in STS4.10 (Tet-On-*anafp*) in presence of 5 and 20 µg/ml Doxycycline. Spores were placed on coverslip surfaces and grown in minimal media for 16 hours at 30 °C. This was followed by treatment with either doxycycline or H2O2 for four hours. Afterwards, cells were fixed with formaldehyde and chromatin condensation was quantified. (A) Shown are exemples of HOECHST-stained *A. niger* STS4.10 after incubation without Doxycycline, with 20 µg/ml Doxycycline, with 5 and 100 mM H_2O_2 (no doxycycline). (B) Box plots display the Nuclear Area Factor (NAF = area x roundness) derived from 10 images containing at least 280 nuclei, each compiled from three separate biological experiments. FW35.1 (complemented progenitor strain) was used as a negative control.

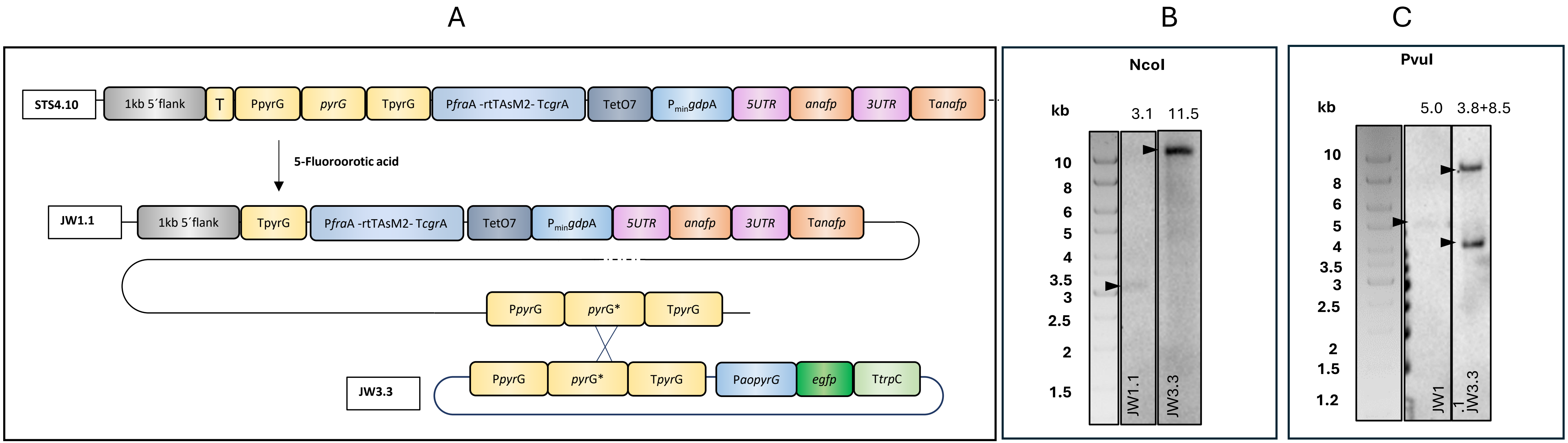

D

Southern Probe:(binds *pyrG*)

CCGTGATCAAAACCCACATCGATATCCTCTCTGATTTCAGCAACGAGACCATTGAGGGACTTAAGGCTCTCGCGCAGAAGCACAACTTTCTCATCTTCGAGGACCGCAAGTTCATTGACATCGGCAACACGGTCCAGAAGCAATACCACG
GCGGTACCCTCCGTATCTCGGAATGGGCCCACATCATCAACTGCAGCATTCTCCCTGGTGAGGGTATCGTCGAGGCTCTCGCTCAGACGGCGTCTGCACCGGACTTCGCCTACGGCCCCGAACGCGGTCTGTTGATCTTGGCAGAGATGA
CCTCTAAGGGCTCCTTGGCTACCGGCCAGTACACTACTTCCTCGGTCGATTATGCCCGGAAATACAAGAACTTCGTTATGGGATTCGTGTCGACGCGCGCGTTGGGTGAGGTGCAGTCGGAAGTCAGCTCTCCTTCGGATGAGGAGGAC
TTTGTGGTCTTCACGACTGGTGTGAACATTTCTTCCAAGGGAGATAAGCTTGGTCAGCAGTACCAGACGCCCGGATCGGCTATCGGCCGGGGTGCTGACTTCATTATCGCGGGTCGCGGTATCTACGCCGCGCCGGATCCGGTGCAGGC
TGC

Figure S9: Cloning strategy and southern blot verification of *A. niger* JW3.3 (Tet-On-*anafp*,+UTR, P*gdpA*-*egfp*). (A) schematic of the *A. niger* STS4.10 (progenitor) *anafp* locus and the 5-FOA counterselected intermediate *A. niger* JW1.1 (*pyrG* auxotroph), resulting in STS19.3 (*pyrG* prototroph) via double recombination. (B, C) Southern blot data of STS4.10 and AB4.1 when cutting with Aat2 and NcoI. Expected band sizes are marked with a black arrowhead and are written above the blot lane in kbp. (D) Southern probe sequence

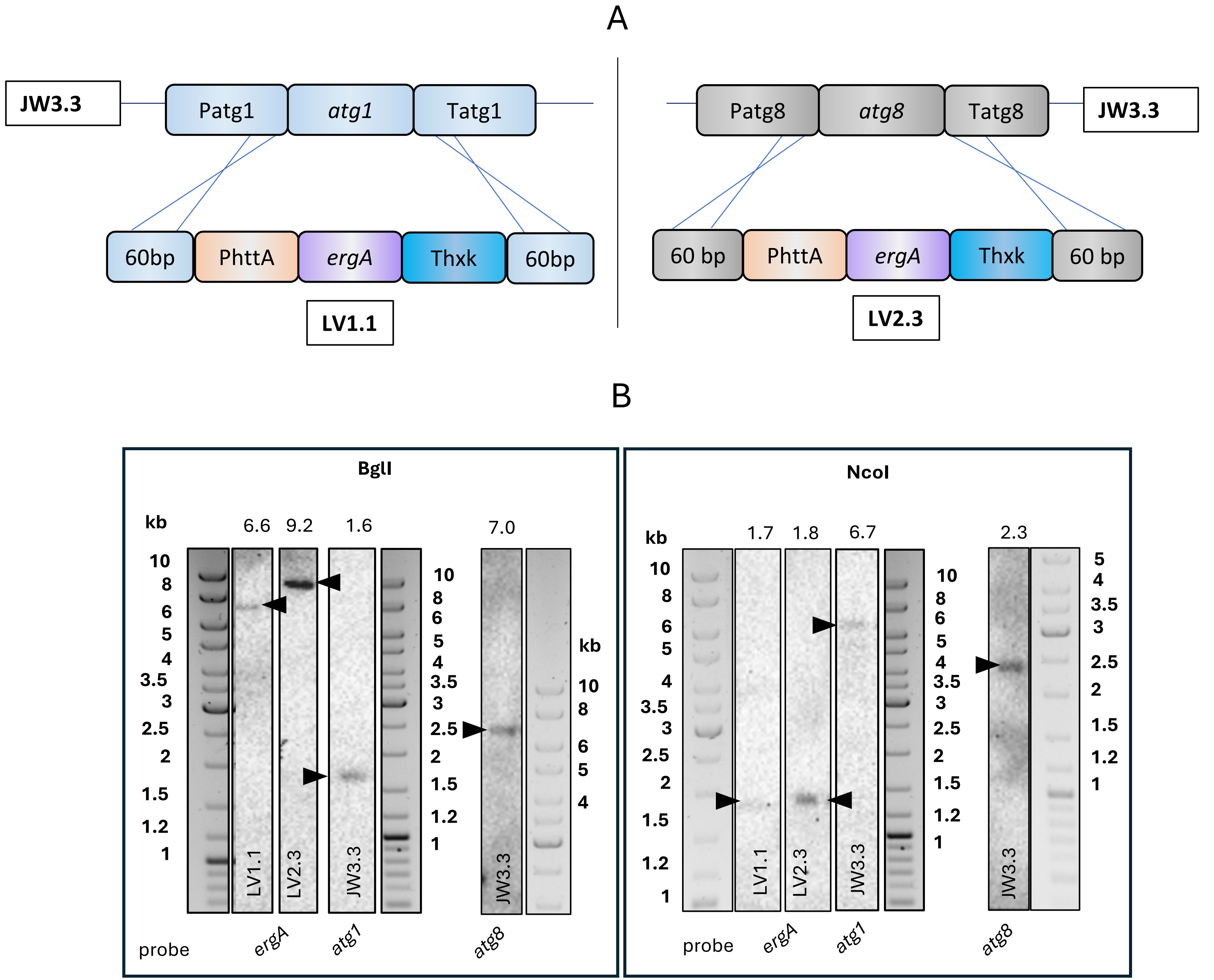

Figure S10: Cloning strategy and southern blot verification of *A. niger* LV1.1 and LV2.3 (Tet-On-*anafp*,+UTR, P*gdpA*-*egfp, Δatg1/ Δatg8*). (A) schematic of the *A. niger* JW3.3 (progenitor) *atg* locus and corresponding donor DNA (*terbinafine resistant*). (B) Southern blot data of LV1.1, LV2.3 and their corresponding progenitor strain JW3.3, when cutting with Bgl1 and NcoI. Expected band sizes are marked with a black arrowhead and are written above the blot lane in kbp.

Southern Probe LV1.1:(binds *atg1 Terminator*)

CGGGGCCAGCTTCTTCTTCTTCTTCTTCTTCTTCTTCTTCCATTCTCCATGCCAGTTCTCTCTGGGGTTTGATATATGCATTGATGCACGAGTGTTTTAGTGTTTCTTCATATGGGTTTATTATAGGGTTGCCGTGATTTGCTCTGTGTTTAT
TGGTTCCCTTGTTCCTTTCCATTTTCTTACAGGATACGCATGCTTTACAGGCCTGTTGGGCGGGTGTTTGCAATTGGCGTTCAAGCTGGGGACCCGGGTACACAAATTGCCAGCGGACATTGGATACGAGTGATGATGATGCCATGGGAT
CATAGGGAGTCAGGGTGGATTCGGGATGGATCTGGAGGATGCAGATACTGCCAGTCAGAGGCAGCTGTACTGTATATACACATAATAACACGCGAATATGTTCATCTCGTGGCGAGGAATGGTGCGCACTGGTTGGAGGATTGCTGCGG
ATTTAGAGCTCGACCCACGGCGGCGGGCATTGGCTCTGGGACCCGCGCAGACCTTAACTTTACCTAACTTAAACGTGCAAGATCCAGGCCCAAATTGCGTGATTGATTTTGGTGTGTCATAGGAATTGCACCGACGGCGTTGAACGGTT
CACCATCTTCTTGGTGTCTGGGCCGTGGTTTGAGAGGTTTGAGAAGTTTGGGCAGGGAGTGGAGAGCTTCCCTCGTTCGGGTTCCGCTCCGACGCCCAGAAGAAGTTTTTTTCTTCTGTACCAAAGAAATTCCCCTTTTGCCTCACGG

Southern Probe LV2.3:(binds *atg8 Terminator*)

GCCGTCATCGTTTCAAGTCACTTCCTTCAGTTGCCCTTTCCTTAATTTGGGGAAGTTGCAGATGAACTTCCTAGATGTGGGTGTTGGTTGCTGTAGGACCTGGATAGATGGCGAGCTCGACCACGCTGTGATGTTTCTATGCTGTGCTGTA
CCCGCGGGCGTTATCTTGGGGGCTCTTCATTAATCTTCGTGTGAACTCTCTTGGCGAAATGAGTATTCTTCAGATGATAGTTTAAATGAGAAACAATCGTTGGCATTTGCTGGAAGCCTGTCTTGAGCTCGTAGATTTAGGTGGATAAGATG
TTCCAGGAGTGCACAAGCTACTACATACTTCCTGGGACCATGGAATAACTAGTTATAATCGAACGAATTAGTAGTTTAGATAACTAACTAATTATCTCAAACCAGTTAGTTTATCACCGGCTGCTGAGATAATCAGGGCCGCTCCGATCCAGG
GCCGGTGGTGCCAGACCCAAAGGGGGCTAGTGGCGTTGAATGCACCGGCCGCGACGCTTGGAGCGGTTCCGAGTCGGCGCAGAAACAGTCAGTCAGTCCATCTTTCTCGGTCTCTTCCTCTTCCTCTTGCTCTTTTCCTTTCCCCCCTCC
CTCTTCCTGCATCCGCCTTCCCTATCATCCTTTTCTGTGCTGCATTGCCTGGCCCCGGCTGTGGTCTCTTGTCCTGTCTTTGTTGGCATTTCTGTCCTATATATAAATCTTCCCGTCCTTCCTCTTTCCTTCTGAATCGCCTTTTCATCTCCCCGAC
GGAAAAGAAGCCTCAGGCCCGGAAAATTTGATACTCGCCAATTCCTGCCCCGACTACGCCCAACTCAGCCCAATTCCCATAATCAAGATGTCTCCCGCTATGGCCCAAGCCGGTGCCGGCTCTGCCGCTAAGGATGTTAAGAGAGAATCC
GCGACTGCTCGTCTTCT

Southern Probe LV1.1/ LV2.3: (binds *ergA resistance gene*)

AGAACGGTGGTGTCAAGGGTTACATGCGAAACAACATCCTGCCCAAGCTCCCAGAGGGTGTCCAGCAGTCGTTCTCCGATGCCCTAGAGAAGGGCCAGCTACGGTCAATGCCGAACTCGTTCCTGCCTGCCTCGGCCAACAAGACACC
CGGCTTGATGATTCTGGGCGATGCGCTTAACATGCGGCACCCTCTCACAGGTGGAGGCATGACTGTGGCGCTCAACGATGTTTGTGTCATCCGCGAATTGCTCAGCCCAGAGCGCGTGCCCAACCTCTCCAATACAGGCCTTGTTCTCGA
GCAGCTCGCAGAGTTCCACTGGAAACGAAAGAACTCATCTTCGGTCATCAATATCCTCGCACAGGCTCTCTATGCGCTCTTCGCTGCAGACAGTACGCACGCTCACTCTTCAGGTTAATTTGCAATAACTAACTGTTCATTCAACAGACTAT
TACCTCAAAGCTCTTCAGCGTGGCTGTTTCCGCTACTTCCAGATTGGACCAGTTGGCGGCCCTGTCGGGTTGTTAGCCGGTCTAATCAAGAAACCCCTCGTCCTTGTCAGCCACTTCTTCTCCGTTGCCTTCCTCGCCATCTGGGTCCACAT
ATGCGATACCCCTCTGTCCAAGCTATTCCTAGCCCCATACTACGCCGTCATGATATTGTACACAGCCTCTGTCGTGATTTTGCCATACATCTGGACCGAGAT

Figure S11: Southern Blot probe sequences used for the generation of LV1.1 and LV2.3

Figure S12: Unmodified southern blot 1 (BBA22.6)

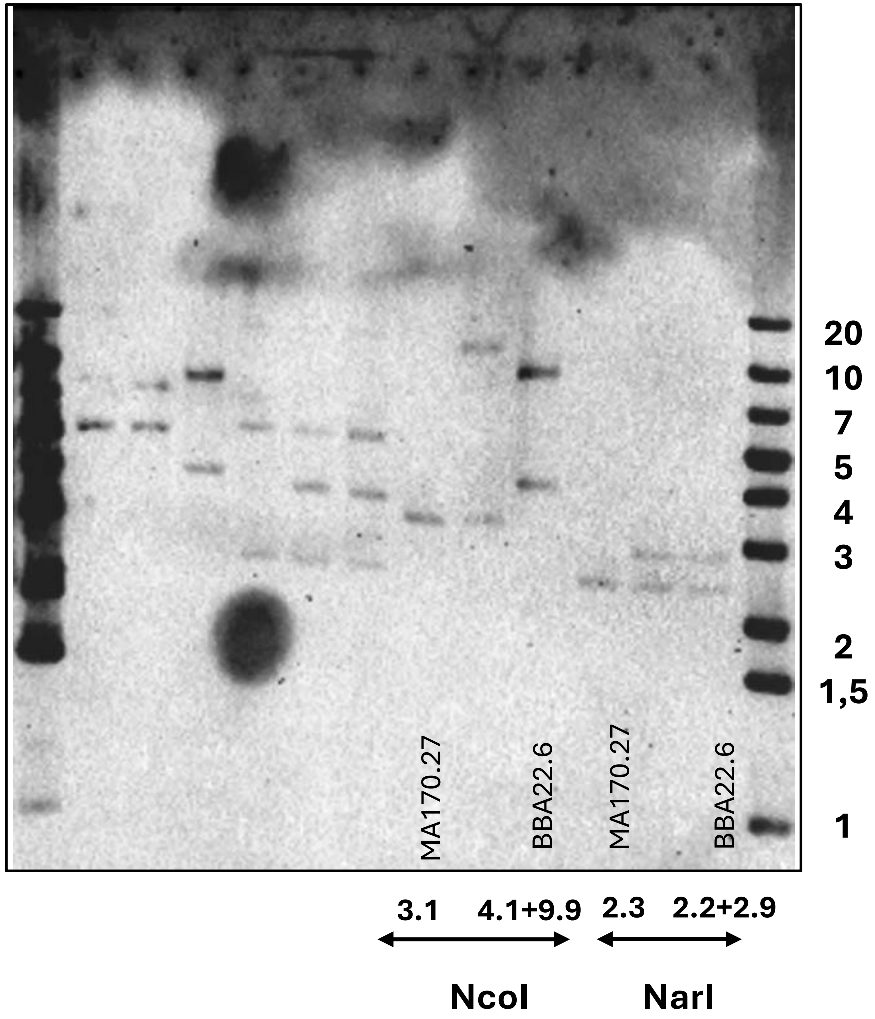

Figure S13: Unmodified southern blot 2 (STS4.10)

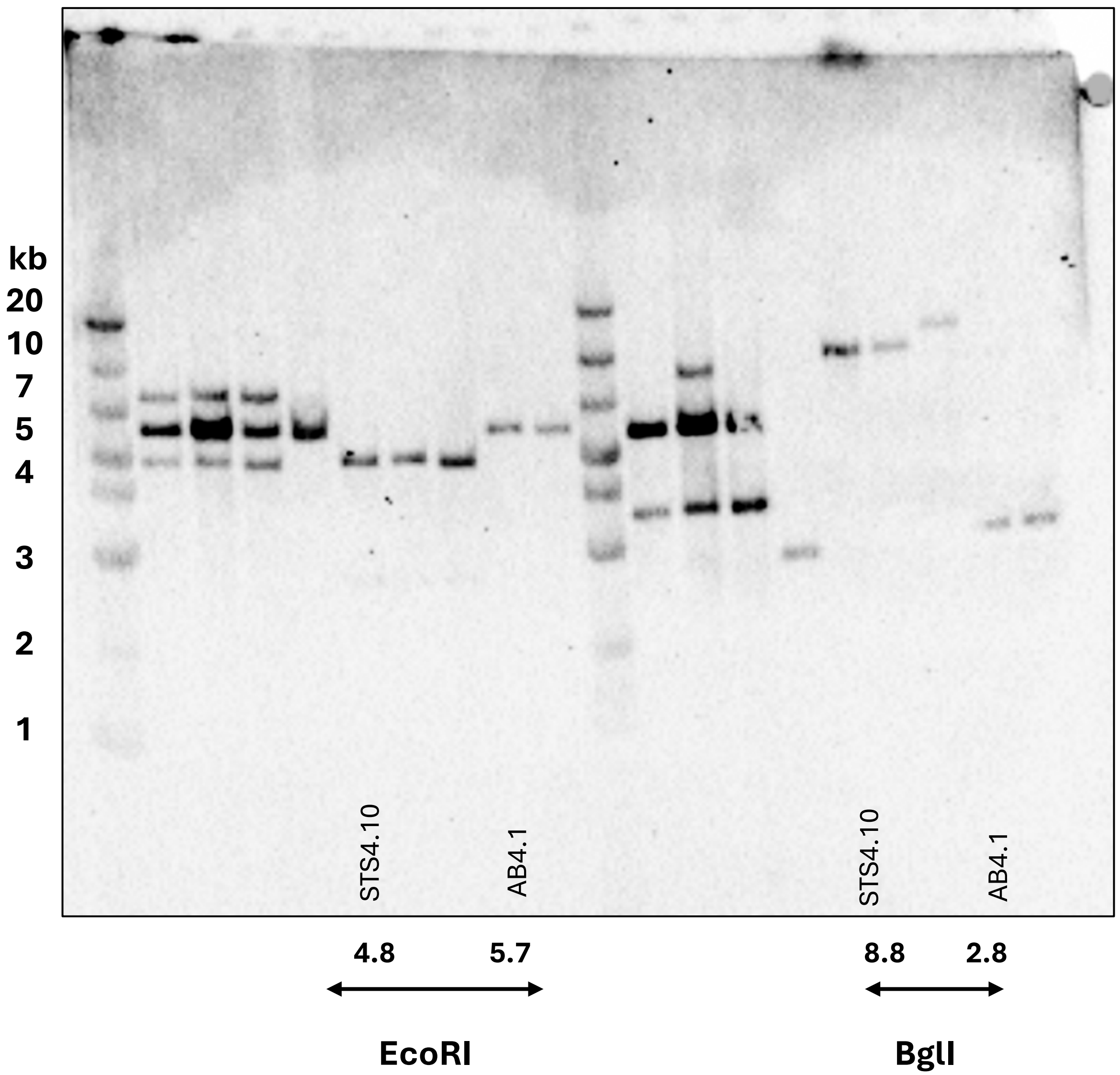

Figure S14: Unmodified southern blot 3 (STS19.3)

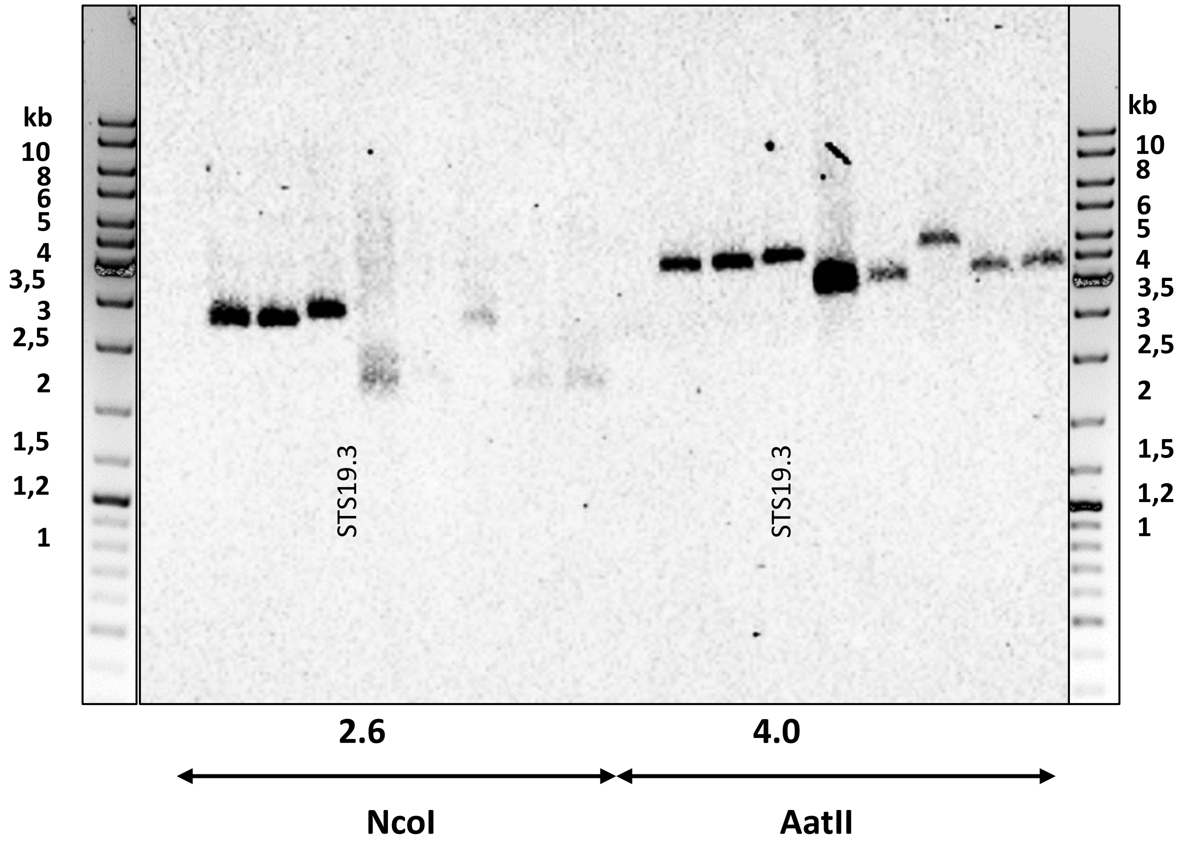

Figure S15: Unmodified southern blot 4 (STS9.2)

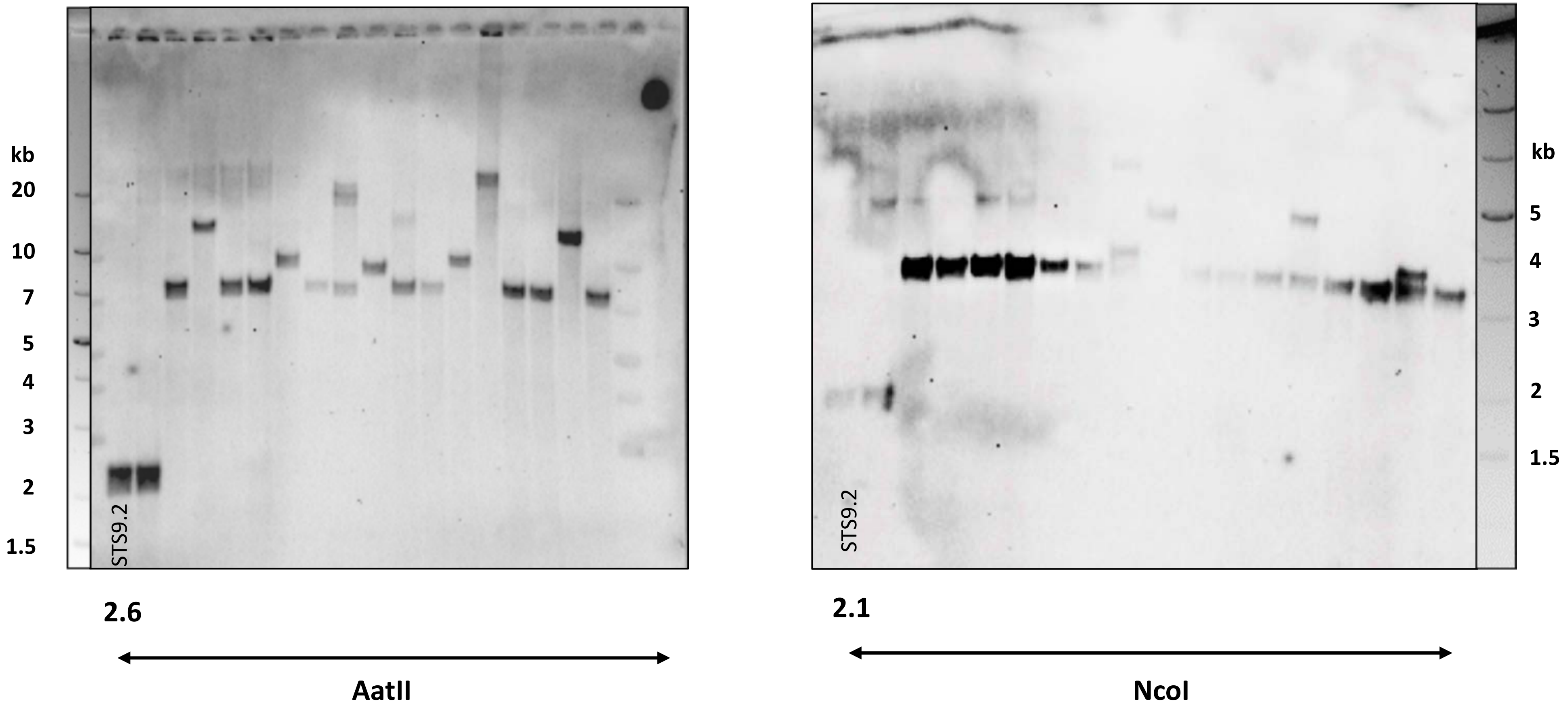

Figure S16: Unmodified southern blot 4 (JW3.3)

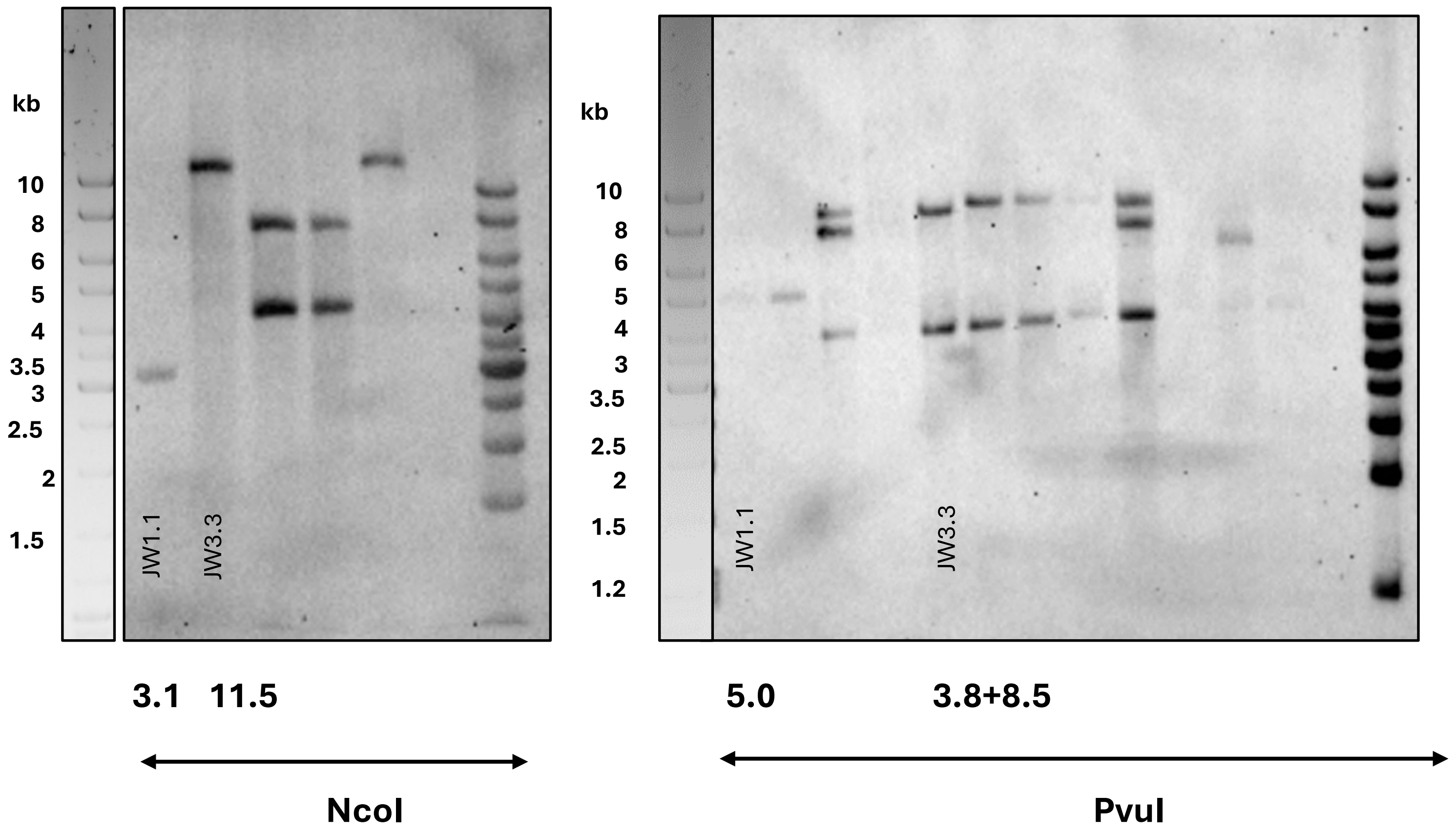

Figure S17: Unmodified southern blot 5 (LV1.1; LV2.3)

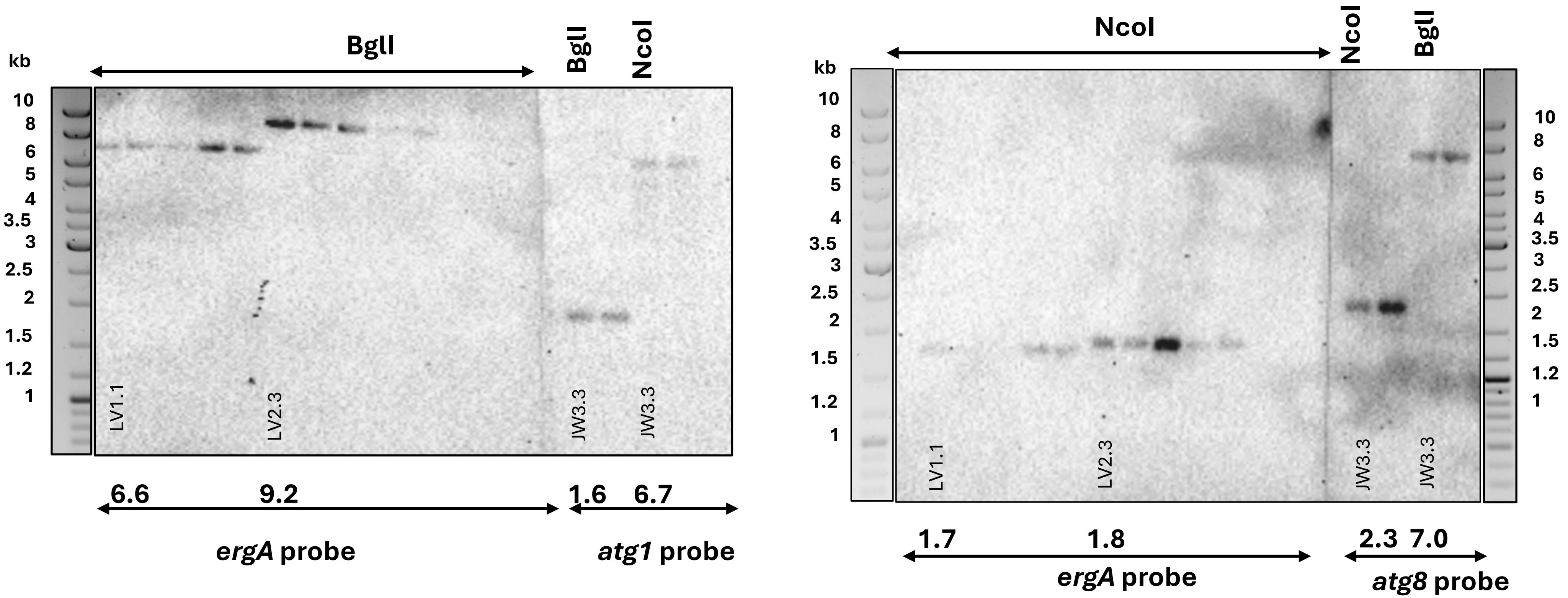

Supplement: Supplementary file 3 [file Data_Sheet_1.zip › Additional File 1 ( Supplementary Figures S1-S17 ).pdf]
